# Effects of Diet and Genetics on Growth Performance of Pigs in Response to Repeated Exposure to Heat Stress

**DOI:** 10.3389/fgene.2017.00155

**Published:** 2017-10-26

**Authors:** Wendy M. Rauw, E. Johana Mayorga, Soi Meng Lei, Jack C. M. Dekkers, John F. Patience, Nicholas K. Gabler, Steven M. Lonergan, Lance H. Baumgard

**Affiliations:** ^1^Departamento de Mejora Genética Animal, Instituto Nacional de Investigación y Tecnología Agraria y Alimentaria, Madrid, Spain; ^2^Department of Animal Science, Iowa State University, Ames, IA, United States

**Keywords:** pigs, selection, heat stress, robustness, resilience, growth, feed efficiency, production

## Abstract

Heat stress (HS) is one of the costliest issues in the U.S. pork industry. Aims of the present study were to determine the consequences of repeated exposure to HS on growth performance, and the effects of a high fiber diet, the genetic potential for high lean tissue accretion, and the genetic potential for residual feed intake (RFI) on resilience to HS. Barrows (*n* = 97) from three genetic lines (commercial, high RFI, low RFI) where subjected three times to a 4-day HS treatment (HS1, HS2, and HS3) which was preceded by a 9-day neutral (TN) adaptation period (TN1) and alternated by 7-day periods of neutral temperatures (TN2, TN3, and TN4). Body weight gain (BWG), feed intake (FI), feed conversion efficiency (FCE), RFI, and the drop in BWG and FI between TN and HS were estimated for each period, and slaughter traits were measured at the end of TN4. Commercial pigs had lower FI when fed a high fiber diet compared to a regular diet (2.70 ± 0.08 vs. 2.96 ± 0.08 kg/d; *P* < 0.05), while no differences were found for BWG, RFI or FCE. HS reduced FI, BWG, and FCE, increased RFI, and resulted in leaner pigs that generate smaller carcasses at slaughter. In TN, commercial pigs grew faster than the low and high RFI pigs (1.22 ± 0.06 vs. 0.720 ± 0.05 and 0.657 ± 0.07; *P* < 0.001) but growth rates were not significantly different between the lines during HS. Growth rates for the low RFI and high RFI pigs were similar both during TN and during HS. Pigs of interest for genetic improvement are those that are able to maintain growth rates during HS. Our results show that response in growth to HS was repeatable over subsequent 4-d HS cycles, which suggests the potential for including this response in the breeding index. The best performing animals during HS are likely those that are not highly superior for growth in TN.

## Introduction

Despite aggressive heat stress (HS) abatement strategies, the U.S. swine industry loses at least $900 million/year to HS (Pollmann, 2010). Sources of reduced revenue include slower growth rates, inconsistent market weights, altered carcass traits, infertility, increased health care costs and mortality. Consequently, HS is currently one of the costliest issues in the U.S. pork industry and compromises the industry's capacity to efficiently produce animal protein for human consumption (Baumgard and Rhoads, 2013). The effect of HS will likely become more of an issue if the frequency of severe hot weather increases as predicted (USDA, 2015). Therefore, there is an urgent need to identify the effects of management practices and genetics on HS induced losses.

A depression in both feed intake and growth rate in HS is a common observation in all heat-stressed livestock (Brown-Brandl et al., 2004). In addition, in some cases a reduced feed efficiency is noted (Brown-Brandl et al., 2000). Commonly used measures of feed efficiency are feed conversion efficiency (FCE) and residual feed intake (RFI). Whereas animals that grow faster have higher FCE, RFI is phenotypically independent of body weight gain but highly correlated with feed intake (Crews, 2005). Therefore, depending on the extent to which HS affects growth and feed intake, it may affect both measurements of feed efficiency differently. Heat stress mediated changes in energy metabolism may result in changes in carcass quality (Pearce et al., 2013).

There are a variety of management strategies to consider during the warm summer months. A nutritional plan may include reducing the amount of dietary fiber because of their large heat increment. Pigs fed high fiber diets are presumably more susceptible to HS (Renaudeau et al., 2012). In addition, there is likely a genotype by environment interaction, implying that high producing genotypes may be more sensitive to HS, such that a different genotype may be more desirable and adaptable to a warmer environment (Rauw and Gomez-Raya, 2015). Animals selected for improved lean tissue accretion produce more metabolic heat and are ostensibly more susceptible to HS (Brown-Brandl et al., 2004). Conversely, selection for reduced RFI (i.e., improved feed efficiency) may reduce metabolic heat production such that feed efficient animals may be more resilient to HS.

Objectives of the present study were to determine the consequences of repeated exposure to HS on body weight gain, feed intake, feed efficiency (FCE and RFI), and carcass quality. Further, we wanted to determine the effects of (a) a high fiber diet; (b) the genetic potential for high lean tissue accretion, and (c) the genetic potential for high feed efficiency on resilience to HS.

## Materials and methods

### Animals

Barrows (*n* = 97) from three genetic lines; a contemporary commercial line (*n* = 31), and pigs from generation 10 of lines divergently selected for low (*n* = 35), and high residual feed intake (RFI; *n* = 31), as described by Cai et al. (2008), were used in this experiment. The contemporary line was a cross between DNA Genetics line 600 Duroc and PIC line Camborough® 22. Barrows from the low and high RFI lines originated from 13 and 10 litters, respectively. Because litter origin was not known for the commercial line, they were assumed to be genetically unrelated. Barrows of the low and high RFI line were weaned at 25–37 days of age (27.4 SD 2.4 d). Because weaning age was not known for commercial barrows, they were assumed to be weaned at the average weaning age of the divergent selection lines for the purpose of statistical analysis. On day 1 of the experiment, the average body weight of the pigs was 59 kg (SD 5.9 kg) in the commercial line, 81 kg (SD 11.0 kg) in the low RFI line, and 81 kg (SD 8.7 kg) in the high RFI line. The experiment was designed such that animals from all three genetic lines would reach slaughter weight at the same time. Thus, given the faster growth rate of the commercial pigs, their starting body weight was considerably lower than that of the low and high RFI lines. On day 1 of the experiment, low and high RFI animals were between 139 and 160 days of age (147 SD 7); the exact age of the pigs of the commercial line was not known. Within each genetic line, pigs were randomly assigned to one of two dietary treatments (low vs. high dietary fiber) arranged in a 2 × 3 factorial design. Pigs were housed individually in one of 54 pens in one of two environmentally controlled rooms. Each pen was equipped with a stainless steel feeder and a nipple drinker. Feed and water were provided *ad libitum* during the entire experiment. All procedures were approved by the Iowa State University Institutional Animal Care and Use Committee, protocol # 2-15-7948S.

### Dietary treatments

Two diets were formulated on a constant SID lysine to net energy ratio within genetic line according to the following specifications: (1) a low fiber diet based on corn-soybean meal (standard finishing diet; 9% NDF), and (2) a high fiber diet containing 25% medium fat (6–8%) corn distillers dried grains with solubles (20% DDGS; 15% NDF). The two diets had similar net energy contents and were formulated to meet or exceed predicted requirements for finishing pigs (National Research Council, 1998) for energy, essential amino acids, protein, minerals and vitamins (Table 1).

**Table 1:** Ingredient inclusion and chemical and nutritional characteristics of experimental diets (as-is-basis).

	**Low Fiber Commercial**	**High Fiber Commercial**	**Low Fiber RFI**	**High Fiber RFI**
Corn (%)	85.33	65.67	87.85	71.00
DDGS (%)	0.00	20.00	0.00	20.00
SBM, 47.7% (%)	11.00	9.70	8.50	7.20
Soybean oil (%)	1.00	2.25	1.00	2.00
Limestone (%)	0.96	1.15	0.96	1.15
Monocalcium Phosphate (%)	0.60	0.20	0.66	0.22
Lysine HCl (%)	0.25	0.23	0.20	0.18
DL Methionine (%)	0.00	0.00	0.00	0.00
L-Threonine (%)	0.05	0.00	0.02	0.00
L-Tryptophan (%)	0.01	0.00	0.01	0.00
L-Valine (%)	0.00	0.00	0.00	0.00
Enzyme (%)	0.00	0.00	0.00	0.00
Vitamin Premix (%)	0.25	0.25	0.25	0.25
Trace Mineral Premix (%)	0.15	0.15	0.15	0.15
Salt (%)	0.40	0.40	0.40	0.40
NE (kcal/kg)	2590	2534	2604	2603
ME (kcal/kg)	3358	3372	3358	3447
NE:ME	0.77	0.75	0.78	0.76
Crude Protein (%)	12.3	15.6	11.3	14.9
ADF (%)	3.04	5.79	2.98	5.81
NDF (%)	8.68	13.53	8.70	13.81
SID AA (%)				
Lys	0.64	0.64	0.54	0.54
Met	0.19	0.24	0.18	0.23
Cys	0.19	0.23	0.18	0.22
Thr	0.41	0.43	0.34	0.40
Trp	0.12	0.12	0.10	0.10
Calcium (%)	0.50	0.50	0.50	0.50
Phosphorus (%)	0.43	0.43	0.43	0.44
STTD Phosphorus (%)	0.23	0.23	0.23	0.23
SAA (%)	0.39	0.47	0.37	0.46

### Experimental design

**Figure 1:**
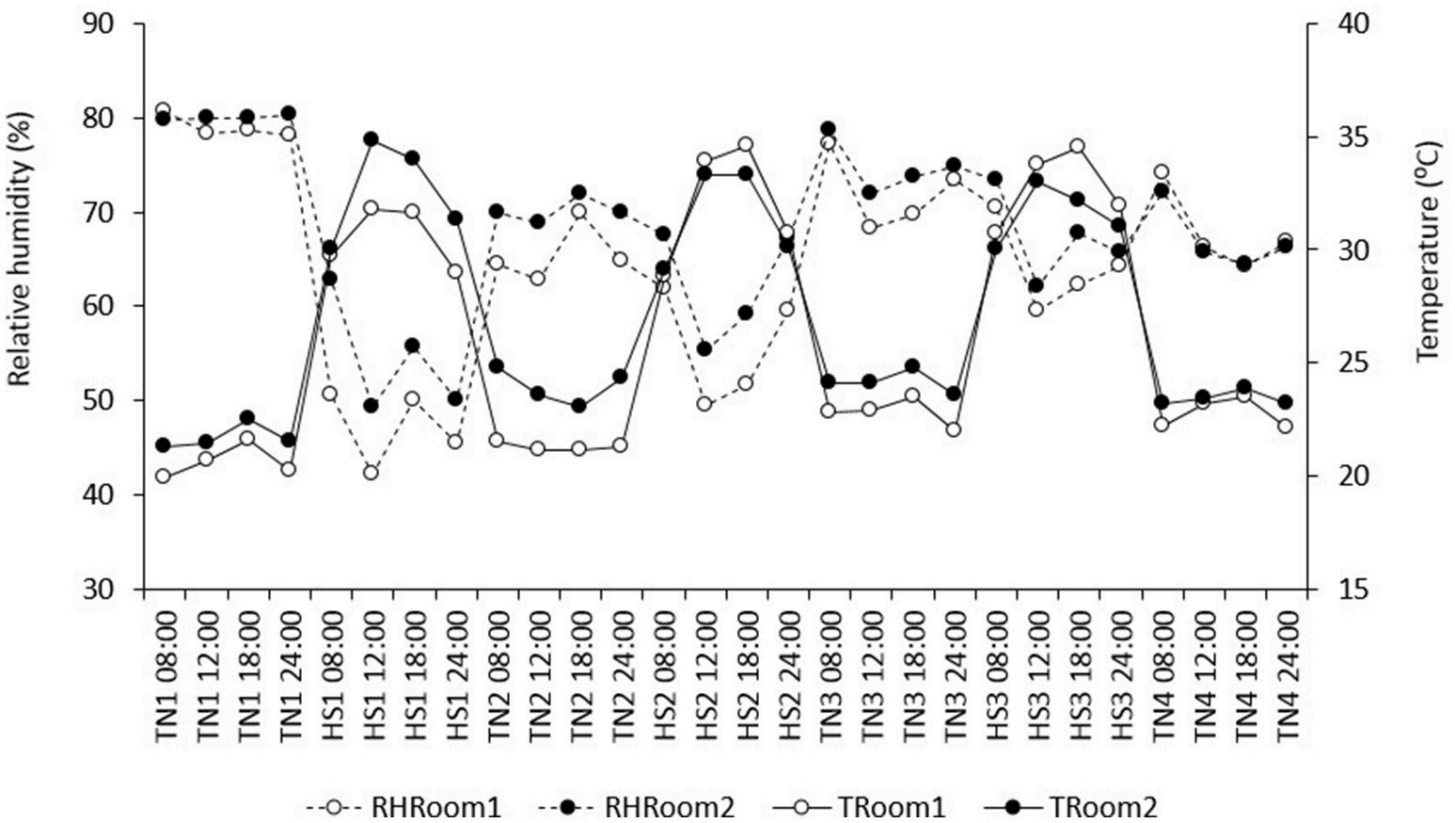
Average relative humidity (RH) and temperature (T) in each period, at 8:00, 12:00, 6:00, and 24:00 h.

The study was divided into seven periods of episodic thermoneutral (TN) and heat stress (HS) conditions, in an attempt to mimic repeated bouts of heat during the summer months: TN1, HS1, TN2, HS2, TN3, HS3, and TN4. During TN1 (20 d), pigs were allowed to acclimate to their respective diets and new environment with a 12 h:12 h light-dark cycle. Subsequently, pigs experienced three periods of HS (HS1, HS2, and HS3), each 4 days in length and each followed by 7 days of intermittent TN conditions (TN2, TN3, and TN4), adding to a total of 52 days. Heat was provided by hanging heaters within the room that were run via a thermostat. Fans were placed throughout the rooms to ensure equal distribution of the environmental conditions throughout the facility, which was verified by four data loggers in each room (Lascar, EL-USB-2-LCD, Erie, PA, USA). Ambient temperature (Ta) and relative humidity (RH) were monitored and recorded every 5 min by the data loggers and then averaged by day. Temperature and relative humidity in each room from day 1 to day 52 are summarized in Figure 1. Average T and RH in TN was 21.9 SD 1.1°C and 71.1 SD 6.1% RH in room 1, and 23.3 SD 1.1°C and 73.1 SD 5.5% RH in room 2. Average T and RH in HS was 31.8 SD 2.1°C and 55.6 SD 8.6% RH in room 1, and 31.8 SD 1.8°C and 61.3 SD 7.5% RH in room 2.

Body weights (BW) were obtained on days 1, 8, and 19 of TN1 and then on the last day of HS1, TN2, HS2, TN3, HS3 and TN4 (days 23, 30, 34, 41, 45, and 52, respectively). Feed intake (FI) was measured daily throughout the experiment as feed disappearance. In addition, 10th-rib back fat thickness (BFT) and loin aye area (LEA) were measured via ultrasound scan at the end of TN1 and TN4 (days 19 and 52, respectively). Three days after the end of TN4 (i.e., on day 55), pigs were slaughtered at a commercial processing plant and hot carcass weight (HCW), loin depth (LoinD) and percentage lean (%Lean) were determined. %Lean was calculated with the Fat O Meater equation as: 58.86 – (BFT × 0.61) + (LoinD × 0.12), as provided by the instrument supplier (SFK Technology A/S, Herlev, Denmark) (Smith et al., 2011).

### Body weight, body weight gain, and feed intake

From the nine BW measurements, body weight gain (BWG) was calculated for each of seven periods (BWGperiod) and expressed in kg/d. Because of logistic reasons, BW was measured 1 day before the start and 1 day before the end of each TN and HS period. Thus, BW on days 20, 24, 31, 35, 42, 46, and 53 were estimated by adding the average BWG in each corresponding TN or HS period to obtain BW at the start of each period. Subsequently, average BWG was estimated for periods TN1 (d 8-20), HS1 (d 20-24), TN2 (d 24-31), HS2 (d 31-35), TN3 (d 35-42), HS3 (d 42-46), and TN4 (d 46-53). In addition, the drop in BWG in kg/d during HS (LossBWG) was calculated as BWG_HS1_-BWG_TN1_, BWG_HS2_-BWG_TN2_, and BWG_HS3_-BWG_TN3_.

Average daily FI was calculated for each of the seven periods (FIperiod) and expressed in kg/d. In addition, the drop in FI in kg/d during HS (LossFI) was calculated as FI_HS1_-FI_TN1_, FI_HS2_-FI_TN2_, FI_HS3_-FI_TN3_.

### Feed efficiency

Two methods were used to quantify feed efficiency: (1) residual feed intake (RFI), and (2) feed conversion efficiency (FCE) calculated as BWG/FI. RFI is defined as the difference between the actual FI and that predicted from a linear multiple regression of FI on maintenance requirements (metabolic body weight, BW^0.75^), BWG, and BFT, and is therefore phenotypically independent of growth rate and size (Koch et al., 1963). In this study, RFI was estimated as the difference between the actual FI of the individual and that expected in a TN environment, as predicted from the average relationship across diets and lines of FI with BW^0.75^, BWG, and BFT in TN, which was based on all individual observations from all three lines in each of the four TN periods (i.e., periods TN1, TN2, TN3, and TN4; following Rauw et al., 2002):

(1) $$\begin{array}{l} {\text{FI}}_{\text{i}(\text{TN})}={\text{b}}_{0(\text{TN})}+({\text{b}}_{1(\text{TN})}\times {{\text{BW}}_{\text{i}}^{0.75}}_{\left(\text{TN}\right)})+({\text{b}}_{2(\text{TN})}\times {\text{BWG}}_{\text{i}(\text{TN})})\\ \;+({\text{b}}_{3(\text{TN})}\times {\text{BFT}}_{\text{i}(\text{TN})})+{\text{e}}_{\text{i}(\text{TN})}, \end{array}$$

where FI_i(TN)_ = daily feed intake of individual i across all TN periods (kg/d), ${\text{BW}}_{\text{i}}^{0.75}$ = metabolic body weight of individual i across all TN periods (kg^0.75^), BWG_i(TN)_ = daily body weight gain of individual i across all TN periods (kg/d), BFT_i(TN)_ = backfat thickness of individual i across all TN periods (mm). b_0(TN)_ is the population intercept for FI in TN, b_1(TN)_, b_2(TN)_, and b_3(TN)_ are the partial regression coefficients representing average maintenance requirements per unit metabolic body weight, average feed requirements for BWG, and average feed requirements related to differences in fatness in TN, respectively; and e_i(TN)_ is the error term, which represents the RFI of individual i in TN in kg/d. Metabolic BW was estimated as the average BW of an individual at the beginning and at the end of each period raised to the power 0.75.

Subsequently, RFI was calculated for each individual in each period, including all individual observations in all three lines in each of the four TN and three HS periods (i.e., periods TN1, HS1, TN2, HS2, TN3, HS3 and TN4):

(2) $$\begin{array}{l} {\text{RFI}}_{\text{i}}={\text{FI}}_{\text{i}}-\{{\hat{\text{b}}}_{0(\text{TN})}+\left({\hat{\text{b}}}_{1(\text{TN})}\times {\text{BW}}_{\text{i}}^{0.75}\right)+\left({\hat{\text{b}}}_{2(\text{TN})}\times {\text{BWG}}_{\text{i}}\right)\\ \;+\left({\hat{\text{b}}}_{3(\text{TN})}\times {\text{BFT}}_{\text{i}}\right)\}, \end{array}$$

where RFI_i_ = RFI of individual i across all TN and HS periods, FI_i_ = average daily feed intake of individual i across all TN and HS periods (kg/d), ${\text{BW}}_{\text{i}}^{0.75}$ = average metabolic body weight of individual i across all TN and HS periods (kg^0.75^), BWG_i_ = average daily body weight gain of individual i across all TN and HS periods (kg/d), BFT_i_ = backfat thickness of individual i (mm), and ${\hat{\text{b}}}_{0\left(\text{TN}\right)}$, ${\hat{\text{b}}}_{1\left(\text{TN}\right)}$, ${\hat{\text{b}}}_{2\left(\text{TN}\right)}$, and ${\hat{\text{b}}}_{3\left(\text{TN}\right)}$ are the estimates of b_1(TN)_, b_2(TN)_, and b_3(TN)_ from model (1). BFT in models (1) and (2) was that taken at the end of TN1 for period TN1 and HS1, at the end of TN4 for period HS3 and TN4, and the average of the two measurements in TN1 and TN4 for periods TN2, HS2 and TN3. Note that RFI_i_ in TN estimated with model (2) equals e_i(TN)_ estimated in model (1). Negative RFI implies a higher efficiency than the average of the population in TN, which was the condition used to estimate average requirements per unit of growth, metabolic body weight and backfat, whereas those with a positive RFI are less efficient. Therefore, RFI during HS gives an estimate of the amount of feed eaten during HS below or above that expected if they would have remained in TN based on the growth, body weight, and fatness of the animal in that HS period.

### Statistical analyses

The effects of line and climate on feed requirements for BWG (i.e., the regression coefficients on BWG) were estimated based on the following mixed model that included all individual observations in all four TN and three HS periods:

(3) $$\begin{array}{l} {\text{FI}}_{\text{i}}={\text{Line}}_{\text{j}}+{\text{Climate}}_{\text{k}}+{\text{Diet}}_{\text{l}}+{\text{Room}}_{\text{m}}+{\text{Litter\{Line}}_{\text{j}}{\text{\}}}_{\text{n}}\\ \;+{\text{ Age}}_{\text{o}}+{\text{BW}}_{\text{i}}^{0.75}\text{+}\left({\text{Line}}_{\text{j}}\times {\text{Climate}}_{\text{k}}\times {\text{BWG}}_{\text{i}}\right)\\ \;+\;{\text{BFT}}_{\text{i}}+{\text{e}}_{\text{i}}, \end{array}$$

were FI_i_ = average daily feed intake of individual i across all TN and HS periods, Line_j_ = effect of genetic line j (fixed effect; commercial, low RFI, high RFI), Climate_k_ = effect of climate k (fixed effect; TN, HS), Diet_l_ = effect of diet l (fixed effect; low fiber, high fiber), Room_m_ = effect of room m (fixed effect; room 1 and 2), Litter{Line_j_}_n_ = effect of litter n nested within line j (random effect), Age_o_ = covariate effect of age o, ${\text{BW}}_{\text{i}}^{0.75}$ = covariate effect of metabolic body weight of individual i across all TN and HS periods, BWG_i_ = covariate effect of body weight gain of individual i across all TN and HS periods, BFT_i_ = covariate effect of backfat thickness of individual i, and e_i_ = error term of animal i across all TN and HS periods.

The SAS program (SAS Inst. Inc., Cary, NC) was used for the statistical analyses of all individual measured, calculated and estimated parameters. The following mixed model with a repeated statement was fitted to describe the data on BW, BWGperiod, FIperiod, RFI, and FCE.

(4) $$\begin{array}{l} {\text{Y}}_{\text{ijklmnop}}=\mu +{\text{Line}}_{\text{i}}+{\text{Climate}}_{\text{j}}+\text{Period}{\left\{{\text{Climate}}_{\text{j}}\right\}}_{\text{k}}+{\text{Diet}}_{\text{l}}\\ \;+{\text{Room}}_{\text{m}}+\text{Litter}{\left\{{\text{Line}}_{\text{i}}\right\}}_{\text{n}}+{\text{Age}}_{\text{o}}\\ \;+{\left(\text{Line}\times \text{Climate}\right)}_{\text{ij}}+{\left(\text{Line}\times \text{Period}\left\{{\text{Climate}}_{\text{j}}\right\}\right)}_{\text{ik}}\\ \;+{\left(\text{Line}\times \text{Room}\right)}_{\text{im}}+{\left(\text{Line}\times \text{Diet}\right)}_{\text{il}}\\ \;+{\left(\text{Diet}\times \text{Climate}\right)}_{\text{lj}}+{\left(\text{Diet}\times \text{Period}\left\{{\text{Climate}}_{\text{j}}\right\}\right)}_{\text{lk}}\\ \;+{\left(\text{Diet}\times \text{Room}\right)}_{\text{lm}}+{\left(\text{Room}\times \text{Climate}\right)}_{\text{mj}}\\ \;{+\text{Room}\times \text{Period}\left\{{\text{Climate}}_{\text{j}}\right\})}_{\text{mk}}+{\text{e}}_{\text{ijklmnop}}, \end{array}$$

where Y_ijklmnop_ = the phenotype measured on animal p, Line_i_ = effect of genetic line i (fixed effect; commercial, low RFI, high RFI), Climate_j_ = effect of climate j (fixed effect; TN, HS), Period{Climate_j_}_k_ = effect of period k nested in climate j (fixed effect), Diet_l_ = effect of diet l (fixed effect; low fiber, high fiber), Room_m_ = effect of room m (fixed effect; room 1 and 2), Litter{Line_i_}_n_ = effect of litter n nested within line i (random effect), Age_o_ = covariate effect of age o (regression coefficient), (Line × Climate)_ij_ = interaction effect between line i and climate j, (Line × Period{Climate_j_})_ik_ = interaction effect between line i and period k (nested within climate j), (Line × Room)_im_ = interaction effect between line i and room m, (Line × Diet)_il_ = interaction effect between line i and diet l, (Diet × Climate)_lj_ = interaction effect between diet l and climate j, (Diet × Period{Climate_j_})_lk_ = interaction effect between diet l and period k (nested within climate j), (Diet × Room)_lm_ = interaction effect between diet l and room m, (Room × Climate)_mj_ = interaction effect between room m and climate j, (Room × Period{Climate_j_})_mk_ = interaction effect between room m and period k (nested within climate j), and e_ijklmnop_ = error term of animal p of genetic line i in climate j, in period k, on diet l, in room m, born in litter n, of age o, e_ijklmnop_~NID(0, $\delta_{\text{e}}^{2}$). LossBWG and LossFI were analyzed with the same model (4) but excluding the effect of climate and its interactions. In addition, LEA and BFT were described by the same model (4) excluding the effect of climate and its interactions but including the covariate effect of BW. Interaction effects in model (4) with a *p*-value of 0.10 and larger were removed from the model (Table 2). Period was identified as the repeated effect in the model for each individual. “Period” corresponded to day 1, 8, 19, 23, 30, 34, 41, 45, and 52, for BW (9 periods), periods TN1 to TN4 for BWGperiod and FI_PERIOD_ (7 periods), periods HS1 to HS3 for LossBWG and LossFI (3 periods), and day 19 and 52 for LEA and BFT (2 periods). The following variance-covariance structures for repeated measures were evaluated to describe individual observations on a trait by trait basis (Table 2): Homogeneous Autoregressive(1) (AR(1)), Heterogeneous Autoregressive(1) (ARH(1)), Compound Symmetry (CS), Toeplitz (TOEP), and Unstructured (UN). The first two models also included the random effect of the individual. Analysis of BW was also evaluated with the spatial power variance components model (sp(pow)), which can be used if observations are not equally spaced in time. Model choice was based on evaluation of fit statistics [the (corrected) Akaike's information criterion and the Sawa Bayesian information criterion], and by using a likelihood ratio test to compare the two best fitting models (provided models were nested) with a chi-square test, using the difference in the −2 Res Log Likelihood and the difference in the number of covariance parameters estimated as test statistics.

**Table 2:** Significance (*p*-values) for line, climate, period, diet, room, age and body weight (BW) and their interactions, on BW, body weight gain (BWGperiod), drop in body weight gain (LossBWG), feed intake (FIperiod), drop in feed intake (LossFI), residual feed intake (RFI), feed conversion efficiency (FCE), loin eye area (LEA), backfat thickness (BFT), percentage lean (%Lean), loin depth (LoinD), and hot carcass weight (HCW).

		**Main Effects**						
	**Model**	**Line**	**Climate**	**Period**	**Diet**	**Room**	**Age**	**BW**
BW^a^	UN	<0.0001	<0.0001	<0.0001	0.6657	0.8091	0.3148	–
BWGperiod^a^	UN	0.0002	0.0275	<0.0001	0.2220	0.0411	0.2833	–
LossBWG^b^	TOEP	0.0006	–	0.0968	0.9519	0.0123	0.2210	–
FIperiod^a^	UN	<0.0001	<0.0001	0.0015	0.0915	0.0001	0.2267	–
LossFI^b^	ARH(1)	0.7427	–	<0.0001	0.1752	0.3403	0.8314	–
RFI^a^	ARH(1)	0.0003	<0.0001	0.0026	0.1758	0.0093	0.4907	–
FCE^a^	ARH(1)	0.9883	0.4519	<0.0001	0.9042	0.0002	0.4413	–
LEA^c^	UN	0.0908	–	0.0060	0.5229	0.2807	0.1488	<0.0001
BFT^c^	UN	<0.0001	–	0.2056	0.3084	0.3429	0.7346	<0.0001
%Lean^e^	MIXED	<0.0001	–	–	0.5331	0.1911	0.7606	–
LoinD^d^	MIXED	0.0165	–	–	0.2410	0.0741	0.0709	0.0166
HCW^d^	MIXED	0.0384	–	–	0.5063	0.2111	0.1343	<0.0001
		**Interaction Effects^f^**						
	**Model**	**L** × **C**	**L** × **P**	**L** × **R**	**L** × **D**	**D** × **P**	**R** × **C**	**R** × **P**
BW^a^	UN	<0.0001	<0.0001	–	–	0.0393	<0.0001	<0.0001
BWGperiod^a^	UN	0.0037	0.0228	–	–	–	0.0096	0.0001
LossBWG^b^	TOEP	–	–	–	–	–	–	0.0294
FIperiod^a^	UN	–	0.0126	0.0008	0.0433	–	–	<0.0001
LossFI^b^	ARH(1)	–	0.0765	–	–	–	–	<0.0001
RFI^a^	ARH(1)	<0.0001	–	0.0718	0.0267	–	0.0155	0.0004
FCE^a^	ARH(1)	0.0099	0.0421	–	–	–	0.0010	<0.0001
LEA^c^	UN	–	0.0003	–	–	–	–	–
BFT^c^	UN	–	0.0512	–	–	–	–	–
%Lean^e^	MIXED	–	–	–	–	–	–	–
LoinD^d^	MIXED	–	–	–	–	–	–	–
HCW^d^	MIXED	–	–	–	–	–	–	–

*L, Line; D, Diet; C, Climate; P, Period; R, Room*.

*Variance-covariance structures used in model (4): Unstructured (UN), Toeplitz (TOEP), Heterogeneous Autoregressive(1) (ARH(1)), and the mixed model used in models (4) and (5) (MIXED)*.

a *Traits analyzed with model (4)*.

b *Traits analyzed with model (4) excluding the effect of climate and its interactions*.

c *Traits analyzed with model (4) excluding the effect of climate and its interactions but including the covariate effect of BW*.

d *Traits analyzed with model (5)*.

e *Traits analyzed with model (5) excluding the covariate effect of BW*.

f *Non-significant interactions were removed from the analyses*.

The following random mixed model was used to evaluate the carcass traits LoinDepth and HCW:

(5) $$\begin{array}{l} {\text{Y}}_{\text{ijklmno}}=\text{μ}+{\text{Line}}_{\text{i}}+{\text{Diet}}_{\text{j}}+{\text{Room}}_{\text{k}}+{\text{Age}}_{\text{l}}+{\text{BW}}_{\text{m}}\\ \;+\;\text{Litter}{\left\{{\text{Line}}_{\text{i}}\right\}}_{\text{n}}+{\left(\text{Line}\times \text{Diet}\right)}_{\text{ij}}+{\left(\text{Line}\times \text{Room}\right)}_{\text{ik}}\\ \;+{\left(\text{Diet}\times \text{Room}\right)}_{\text{jk}}+{\text{e}}_{\text{ijklmno}}, \end{array}$$

where BW_m_ = covariate effect of body weight m, and all other effects are as given in model (4). In addition, %Lean was analyzed with the same model (5), but excluding the effect of BW. Interaction effects in model (5) with a *p*-value of 0.10 and larger were removed from the model (Table 2).

Results are presented as least squares means adjusted for the effects in models 4 and 5. Partial correlation coefficients among traits were estimated after correcting the phenotypes for the effects of line, diet, and room.

## Results

### Body weight and body weight gain

**Figure 2:**
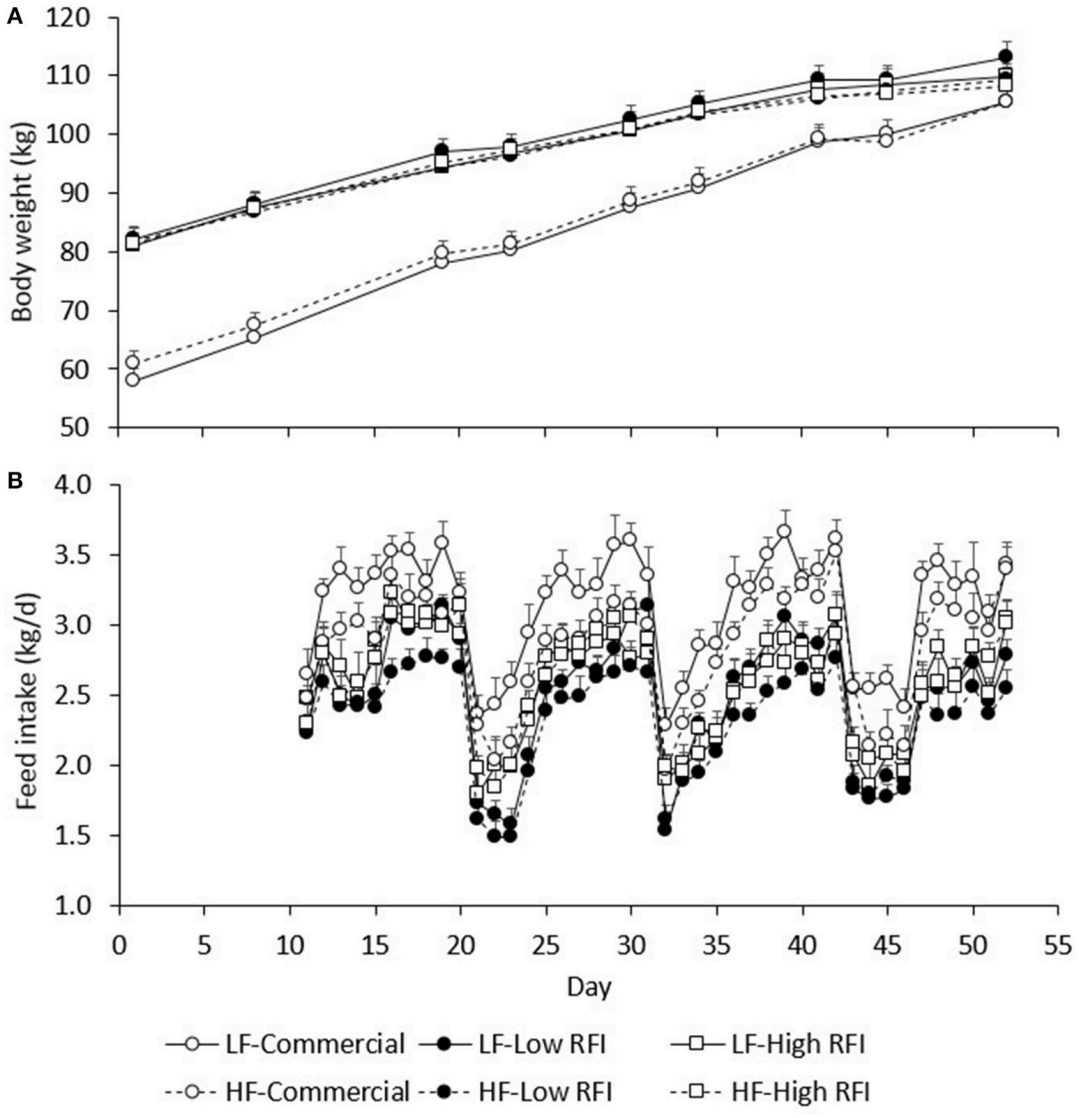
Average body weight **(A)** and average daily feed intake **(B)** between day 1 and 52 of the experimental period, by line and by diet. LF, Low fiber; HF, High Fiber.

Figure 2A presents BW during the experiment for each line and each diet. Body weights of pigs fed the low fiber diets were not significantly different from those fed the high fiber diets. By design, commercial pigs were lighter than the low and high RFI pigs until the end of the experiment (*P* < 0.001).

**Figure 3:**
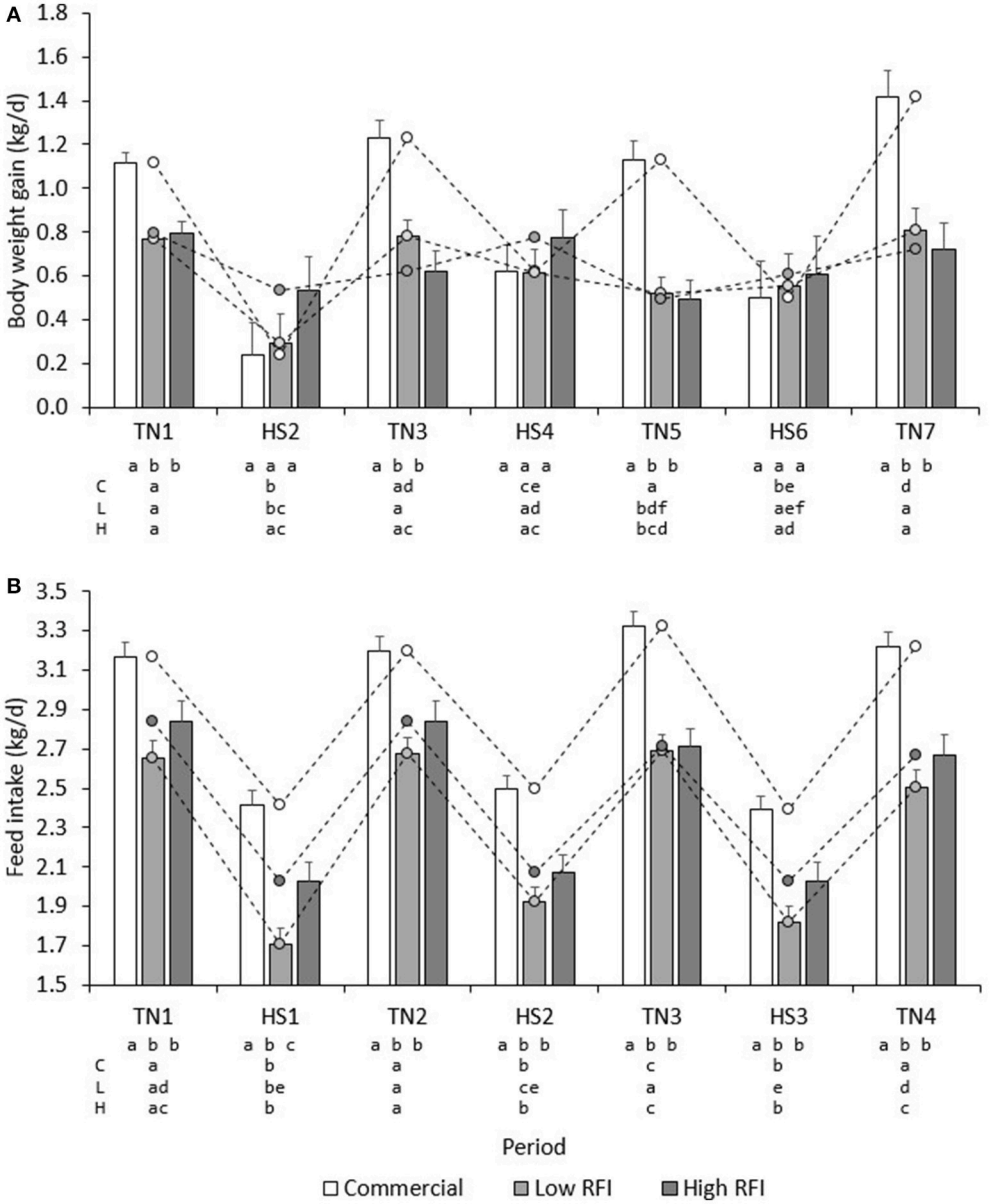
Least squares means (± s.e.) of daily BWG **(A)**, and FI **(B)** for each line in each period. a, b, c, d first line: bars within period between lines with a different letter differ; a, b, c, d second, third and fourth line: bars within line between periods with a different letter differ; C, commercial line; L, Low RFI line; H, High RFI line.

Figure 3A presents BWG (kg/d) for each line in each period. No significant differences existed between diets. Overall, BWG in HS was lower than in TN for the commercial (0.451 ± 0.11 vs. 1.22 ± 0.058 kg/d), low RFI (0.489 ± 0.092 vs. 0.720 ± 0.053 kg/d) and high RFI pigs (0.638 ± 0.11 vs. 0.657 ± 0.065 kg/d), but this difference was significant only for the commercial line (*P* < 0.0001) and suggestive for the low RFI line (*P* = 0.08). In TN, commercial pigs grew faster than the low and high RFI pigs (*P* < 0.001) but growth rates were not significantly different between the lines in HS. Growth rates for the low RFI and high RFI pigs were similar both in TN and in HS.

BWG was positively correlated across TN environments, and this was significant between TN2, and TN3 (*r* = 0.34, *P* < 0.01) and TN4 (*r* = 0.37, *P* < 0.05), and between TN3 and TN4 (*r* = 0.63, *P* < 0.0001). Animals with a higher BWG in HS1 also had a higher BWG in HS2 (*r* = 0.39, *P* < 0.01), and animals with a higher BWG in HS2 also had a higher BWG in HS3 (*r* = 0.51, *P* < 0.001). Interestingly, BWG in consecutive TN and HS periods were negatively correlated: animals with a higher BWG in periods TN1, HS1, TN2, HS2, TN3, and HS3, had a lower BWG in the following periods HS1, TN2, HS2, TN3, HS3, and TN4, respectively (*r* = −0.31 to −0.80, *P* < 0.01).

**Figure 4:**
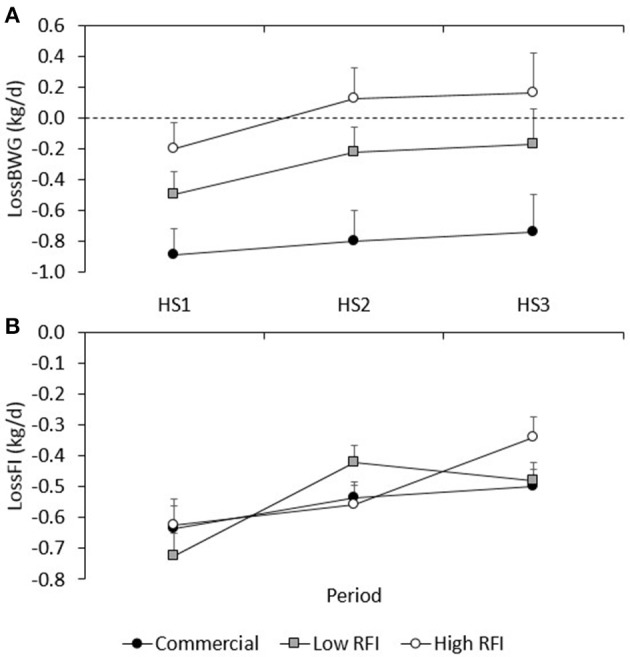
Least squares means (± s.e.) of the absolute drop in body weight gain **(A)**, and feed intake **(B)** between TN and HS environments for each line in periods HS1, HS2, and HS3.

Figure 4A shows the LossBWG between TN and HS environments for periods HS1, HS2, and HS3 for each line. No significant differences existed between diets. Overall, the drop in BWG was larger in HS1 (−0.529 ± 0.092 kg/d) than in HS2 (−0.294 ± 0.11 kg/d) and in HS3 (−0.245 ± 0.17 kg/d) (*P* < 0.05), and larger in the commercial line (−0.774 ± 0.14 kg/d) than in the low (−0.306 ± 0.13 kg/d) and high RFI lines (0.012 ± 0.15 kg/d). Animals that had a larger drop in HS1 also had a larger drop in HS2 (*r* = 0.36, *P* < 0.001), and animals that had a larger drop in HS2 also had a larger drop in HS3 (*r* = 0.51, *P* < 0.001).

**Figure 5:**
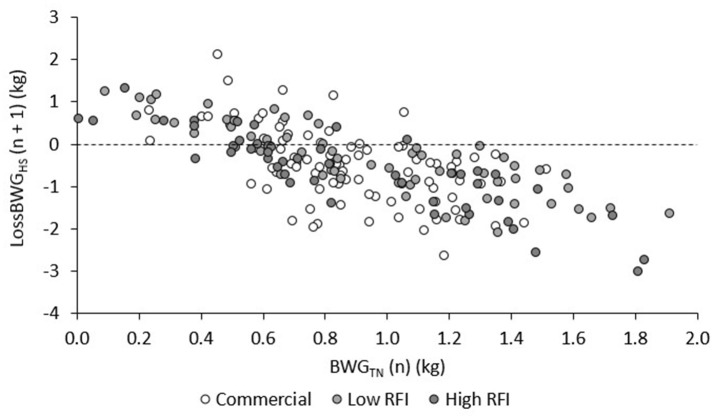
Correlations between BWG in periods TN1, TN2, and TN3 (n) and LossBWG in the subsequent (n + 1) period HS1, HS2, and HS3, for each line.

The relationship between BWG in TN and the subsequent LossBWG in HS is provided in Figure 5, for each genetic line. After adjustment for line, diet, and room, animals that grew faster in TN had a larger drop in BWG in the subsequent HS period (*r* = −0.53, −0.81, and −0.88 for HS1, HS2, and HS3, respectively; *P* < 0.0001). Taking all values together and adjusting them for period, this resulted in a negative and highly significant correlation between BWG in TN and LossBWG in the subsequent HS period (*r* = −0.70, *P* < 0.0001).

### Feed intake

Figure 2B presents the daily FI recorded during the experiment. Results show an upward trend in FI during the first days of TN2 and TN3 following HS1 and HS2, respectively. Figure 3B presents the least squares means of daily FI (kg/d) for each line in each period. Commercial pigs fed the low fiber diet ate more than commercial pigs fed the high fiber diet (2.96 ± 0.079 vs. 2.70 ± 0.076 kg/d; *P* < 0.05), but this difference was suggestive only for the low RFI line (2.28 ± 0.080 vs. 2.17 ± 0.085 kg/d; *P* = 0.08) and was not significant for the high RFI line (2.36 ± 0.091 vs. 2.45 ± 0.096 kg/d). Overall, FI in HS was lower than FI in TN for the commercial (2.44 ± 0.056 vs. 3.22 ± 0.064 kg/d), low RFI (1.82 ± 0.072 vs. 2.63 ± 0.078 kg/d) and high RFI pigs (2.04 ± 0.082 vs. 2.77 ± 0.089 kg/d) (*P* < 0.0001). Commercial pigs ate more than pigs of both RFI lines in all periods (*P* < 0.05), while pigs of the high RFI line ate more than pigs of the low RFI line in period HS1 (*P* < 0.05). FI in each period was significantly positively correlated with FI in all other periods (*r* = 0.31–0.78, *P* < 0.0001).

Figure 4B gives the absolute LossFI between TN and HS environments for HS1, HS2, and HS3, for each line. No significant differences in LossFI existed between diets. Overall, the drop in FI was larger in HS1 (−0.662 ± 0.045 kg/d) than in HS2 (−0.506 ± 0.033 kg/d) and HS3 (−0.439 ± 0.034 kg/d) (*P* < 0.01); differences in LossFI between lines were not significant. Animals that had a larger drop in FI in HS1 also had a larger drop in HS3 (*r* = 0.30, *P* < 0.01).

### Feed efficiency

**Figure 6:**
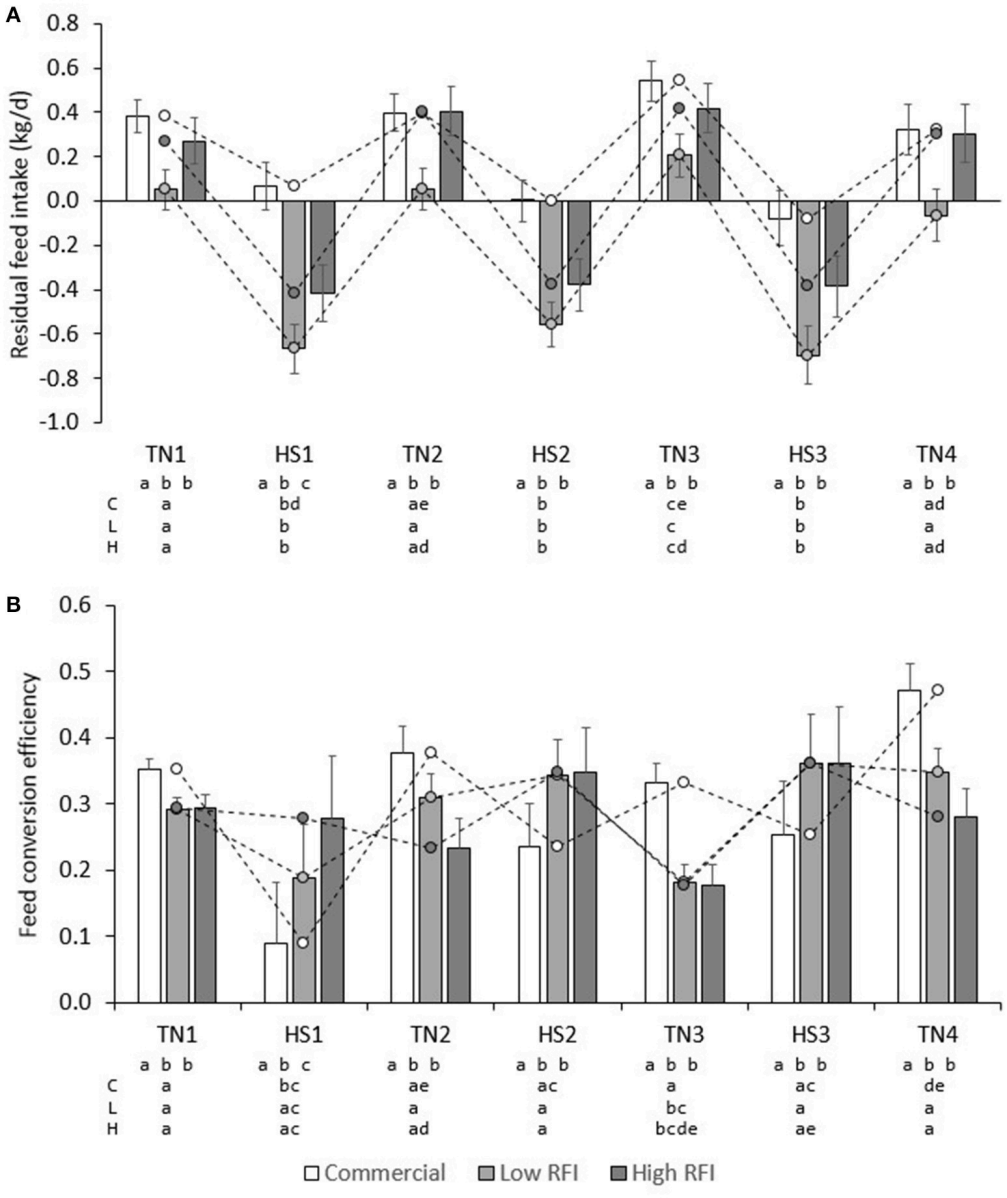
Least squares means (± s.e.) of daily residual feed intake **(A)**, and BWG/FI **(B)** for each line in each period.

**Figure 7:**
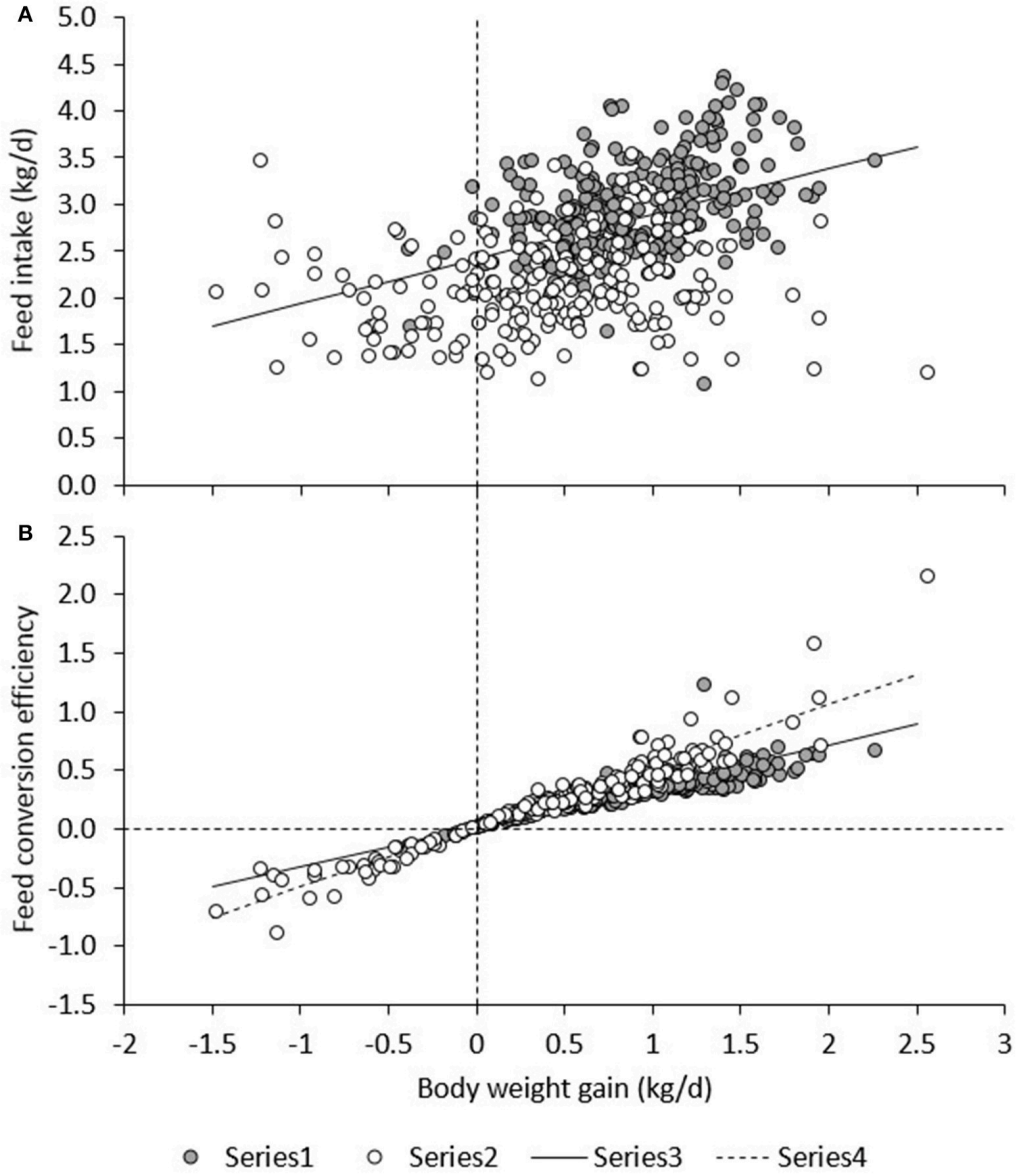
Relationship between BWG, and FI **(A)** and BWG/FI **(B)** under heat stress (HS) and in a thermoneutral environment (TN). The regression line (Regr) is given for TN in **(A)** and for TN and HS in **(B)**.

Figure 6A presents daily RFI for each line in each period. Model (1), which was based on measurements in the TN environment only, had an *R*^2^ of 19%; the intercept (2.42 ± 0.26) and contribution of BWG (0.480 ± 0.045) were significant (*P* < 0.0001), but not the contribution of BW^0.75^ (−0.01 ± 0.01) or BFT (0.00 ± 0.01). For observations in the HS environment only, the *R*^2^ of Model (1) was only 3%, and estimates of regression coefficients were significantly different (*P* < 0.05) from those estimated in TN; the intercept (2.09 ± 0.37) and the contribution of BFT (−0.016 ± 0.01) were significant (*P* < 0.05), but not the contribution of BWG (0.0697 ± 0.052) or BW^0.75^ (0.01 ± 0.01). Figure 7A presents the relationship between BWG and FI in HS and TN, and the regression line corresponding to the intercept and the contribution of BWG_TN_ to variation in FI_TN_ according to model (1). Although the true regression is three-dimensional, including BW^0.75^ and BFT in addition to BWG, Figure 7A visualizes the concept of RFI: animals below the regression line have a negative RFI and are more feed efficient than those above the regression line.

Low RFI pigs fed the low fiber diet had higher RFI (−0.174 ± 0.097) than low RFI pigs fed the high fiber diet (−0.385 ± 0.099), but RFI was not significantly different between pigs fed high or low fiber diets in the commercial line (0.293 ± 0.095 vs. 0.114 ± 0.10 kg/d) and the high RFI line (−0.100 ± 0.11 vs. 0.033 ± 0.11 kg/d). RFI in HS was lower than RFI in TN in commercial (−0.007 ± 0.082 vs. 0.413 ± 0.071 kg/d), low RFI (−0.629 ± 0.094 vs. 0.069 ± 0.088 kg/d) and high RFI pigs (−0.401 ± 0.107 vs. 0.333 ± 0.100 kg/d) (*P* < 0.0001). In addition, overall pigs had higher RFI in TN3 than in TN1, TN2, and TN4 (*P* < 0.05) (Figure 6A). In TN, RFI was significantly lower for the low RFI pigs than for the commercial (*P* < 0.0001) and high RFI pigs (*P* < 0.05). In HS, RFI was higher for the commercial pigs than for both low and high RFI pigs (*P* < 0.001), but differences in RFI between the low and high RFI pigs were no longer significant (*P* = 0.10). Pigs with higher RFI in TN1 also had a higher RFI in TN2 (*r* = 0.57, *P* < 0.0001), but a lower RFI in TN4 (*r* = −0.30, *P* < 0.01). Pigs with a higher RFI in TN4 also had a higher RFI in TN2 and TN3 (*r* = 0.26 and 0.03, respectively; *P* < 0.05). Pigs with a higher RFI in HS2 also had a higher RFI in HS3 (*r* = 0.76, *P* < 0.0001). Pigs with a higher RFI in TN1 also had a higher RFI in HS1, HS2, and HS3 (*r* = 0.73, 0.50, and 0.24, respectively; *P* < 0.05), pigs with a higher RFI in TN2 also had a higher RFI in HS2 (*r* = 71, *P* < 0.0001), but pigs with a higher RFI in HS1 had a higher RFI in TN2 (*r* = 0.71, *P* < 0.0001) but a lower RFI in TN4 (*r* = −0.50, *P* < 0.0001).

The effects of line and climate on feed requirements for BWG as estimated with model (3) are given in Table 3. Feed requirements for BWG were higher in TN than in HS, highest for the commercial line and lowest for the low RFI line. Feed requirements per unit BWG were higher in TN than in HS in the low and high RFI lines. The relationship between RFI in TN and the subsequent LossBWG in HS, after adjustment for line, diet, and room was not significant (*r* = −0.12, 0.14, and −0.10 in HS1, HS2, and HS3, respectively).

**Table 3:** Estimates of the effects of line and climate on the feed requirements for BWG based on model (3), for each line.

**Effect**	**Climate**	**Diet**	**Line**	**Room**	**Estimate**	**s.e**.
Intercept					1.95	0.91
Climate	TN				0.69	0.06
Climate	HS				0	–
Diet		High Fiber			0.07	0.04
Diet		Low Fiber			0	–
Line			Commercial		0.40	0.14
Line			Low RFI		−0.26	0.15
Line			High RFI		0	–
Room				1	0.16	0.05
Room				2	0	–
Age					−0.02	0.03
MBW					0.006	0.008
BWG × C × L	TN		Commercial		0.18	0.06
BWG × C × L	TN		Low RFI		0.25	0.07
BWG × C × L	TN		High RFI		0.21	0.08
BWG × C × L	HS		Commercial		0.21	0.07
BWG × C × L	HS		Low RFI		0.07	0.05
BWG × C × L	HS		High RFI		0.07	0.06
BackFat					0.01	0.01

*C, climate; L, line; TN, thermoneutral environment; HS, heat stress environment; RFI, residual feed intake; BWG, body weight gain (kg/d); MBW, metabolic body weight (kg^0.75^)*.

Figure 6B presents FCE for each line in each period. FCE was not significantly different between pigs fed high or low fiber diets. For commercial pigs, FCE was lower in HS (0.193 ± 0.055) than in TN (0.383 ± 0.021; *P* < 0.01), but FCE was not significantly different between TN and HS for the low (0.298 ± 0.049 vs. 0.283 ± 0.020) and high RFI lines (0.329 ± 0.057 vs. 0.246 ± 0.024). Figure 6B shows an irregular pattern for FCE in TN vs. HS in the low and high RFI pigs. Animals with higher FCE in one period also had higher FCE in any other period (*r* = 0.25–0.76, *P* < 0.05). Figure 7B presents the relationship between FCE and BWG. When animals grew faster, the amount of BWG per unit FI (i.e., their efficiency) increased. This increase was greater during HS (0.517 ± 0.0094) than during TN (0.334 ± 0.0081; *P* < 0.0001).

### Loin eye area, backfat thickness, and slaughter traits

Results for LEA and BFT at TN1 and TN4, and for LoinD, HCW, and Lean% at slaughter are given in Table 4, for each line. The first four traits were adjusted for the effect of BW. None of these traits were significantly different between pigs fed high vs. low fiber diets. Even after correction for BW, LEA was overall larger in TN4 (38.4 ± 0.652 cm^2^) than in TN1 (35.7 ± 0.571 cm^2^; *P* < 0.01). In TN1, LEA was smaller in commercial pigs than in the low and high RFI pigs (*P* < 0.05), but differences between lines were no longer significant in TN4. Commercial pigs had lower BFT than the low and high RFI pigs, both at TN1 and at TN4. After correction for BW, low RFI pigs had a larger LoinD than commercial and high RFI pigs (*P* < 0.05). Commercial pigs had lower HCW than low RFI (*P* < 0.05) pigs, with high RFI pigs intermediate. Commercial pigs had a higher lean percentage than the low and high RFI pigs (*P* < 0.0001).

**Table 4:** Least squares means (± s.e.) of loin eye are (LEA) and backfat thickness (BFT) at TN1 and TN4, and loin depth (LoinD), hot carcass weight (HCW), and Lean% at slaughter, for each line.

	**Commercial**	**Low RFI**	**High RFI**
LEA TN1 (cm^2^)	33.3 ± 1.02	36.6 ± 0.660	37.3 ± 0.728
LEA TN4 (cm^2^)	38.2 ± 0.875	39.0 ± 0.942	38.1 ± 0.989
BFT TN1 (mm)	13.5 ± 0.998	17.6 ± 0.784	19.3 ± 0.896
BFT TN4 (mm)	14.0 ± 0.850	20.0 ± 1.03	19.9 ± 1.13
LoinD (mm)	55.6 ± 1.03	59.0 ± 1.01	54.8 ± 1.11
HCW (kg)	83.8 ± 0.960	87.5 ± 1.01	85.8 ± 1.17
Lean% (%)	56.5 ± 0.461	52.7 ± 0.495	52.1 ± 0.578

The partial correlation between lossFI averaged over periods HS1, HS2, and HS3, and BFT at TN1 and TN4, adjusted for the effects of line, diet, and room, was positive (*r* = 0.19, *P* = 0.06 vs. *r* = 0.23, *P* < 0.05, respectively), while the correlation with Lean% was negative, although not significant (*r* = −0.14, *P* = 0.18). This shows that animals with a lower drop in FI during HS, were fatter. In other words, pigs with a larger drop in FI during HS became leaner. In addition, animals that had a lower drop in FI tended to have larger LoinD (*r* = 0.18, *P* = 0.10) and had heavier hot carcass weights (*r* = 0.26, *P* < 0.05). The correlation between the partial correlation coefficients of lossBWG averaged over periods HS1, HS2, and HS3 with BFT, Lean%, and LoinD was not significant. In addition, the correlation of average lossFI and lossBWG with LEA was not significant.

## Discussion

### Effect of heat stress on feed intake, growth and slaughter traits

Animals are heat-stressed when environmental temperatures are higher than their thermal comfort zone and when thermoregulatory, physiological and behavioral corrections that are designed to remain euthermic are invoked above normal maintenance needs to maintain body temperature, which can negatively impact animal welfare and reduce profitable production. The upper critical temperature of the thermal comfort zone is determined by the balance between external heat load, internal heat production, and heat dissipation. Beyond this point, the animal needs to resort to behavioral and physiological coping mechanisms to eliminate additional heat load or reduce heat production. For example, Aarnink et al. (2006) showed that pigs chose to lie on a cooler floor surface when temperatures increased above approximately 20°C for pigs of 100 kg and above approximately 25°C for pigs of 25 kg. This pattern in response to increasing ambient temperatures can be described by a broken line model, indicating a threshold above which pigs resort to behavioral coping mechanisms. Based on this broken line model, (different) inflection temperatures can also be established for various physiological responses, including respiration rate (Huynh et al., 2005; Banhazi et al., 2008). Although pigs can tolerate a mild heat load, severe HS and/or prolonged periods of HS will eventually result in distress and negatively impact animal welfare (Curtis, 1983).

External factors that determine environmental heat load include air temperature, relative humidity, velocity of ambient air, shade, stocking density, the degree of solar radiation, and conductive and convective heat loss and gain; internal factors that determine susceptibility to HS include the animals' genetic make-up, size (age), insulation, physiological status, and physiological and behavioral plasticity of the coping response. For example, older, larger pigs have a broader thermoneutral zone and a lower upper critical temperature than younger, lighter pigs (Schrama et al., 1996; Quiniou et al., 2000). In particular, internal heat production is determined by processes that regulate metabolic rate, i.e., it is a combination of the oxidation of feed energy to sustain pre-absorptive basic processes necessary to sustain life (basic metabolic rate), post-absorptive processes and spontaneous low levels of activity (resting metabolic rate), medium levels of activity performed during days or weeks (sustained metabolic rate) and short bursts of high energy demanding activities (maximum metabolic rate) (Naya and Bacigalupe, 2009). Pigs selected for increased lean tissue accretion rates have lower upper critical temperatures because the heat associated with protein synthesis and turnover is high compared to the heat associated with synthesizing and maintaining adipose tissue (Millward and Garlick, 1976; Brown-Brandl et al., 2004). Likewise, lactation markedly increases metabolic rates (Eissen et al., 2000). In lactating dairy cattle, Berman and Meltzer (1973) estimated that each increase in 10 kg fat-corrected milk produced per day reduced the upper critical temperature by about 4°C.

During HS, deployed thermoregulatory mechanisms are designed to promote body heat loss. This involves an increase in pulmonary ventilation, respiration rate, and heart rate, therefore, HS is thought to increase basal metabolic rate (Saxton, 1981). When heat dissipation is maximum and the metabolic rate related with activity has been reduced to resting levels, there is no other option than to reduce metabolic functions to further decrease heat production in order to maintain thermal homeostasis. For example, heat stressed lactating sows have been shown to reduce both feed intake and milk production to such an extent that the upper critical temperature for lactating and non-lactating sows was found to be similar in the study of Black et al. (1993). Results of the present study show a profound depression in both feed intake and growth rate in HS, which is a common observation in all heat-stressed livestock (e.g., Kadzere et al., 2002; Brown-Brandl et al., 2004; Lara and Rostagno, 2013). Le Dividich et al. (1998) reported that, depending on animal characteristics, environmental conditions, and experimental design, feed consumption dropped by 40–80 g/d per°C increase in ambient temperature between 20 and 30°C. Renaudeau et al. (2011) reported that the effect of ambient temperature is particularly affected by the BW of the pig, such that the decline in FI between 20 and 30°C averages 32 g/d per°C at a BW of 50 kg and 78 g/d per°C at a BW of 100 kg. Lopez et al. (1991) reported a 10.9% reduction in FI in pigs kept at a hot diurnal temperature between 22.5 and 35°C compared to pigs kept at a constant thermoneutral temperature of 20°C. In the study of Hyun et al. (1998), feed intake dropped by 7.4% in pigs with an initial body weight of 34.7 kg when ambient temperatures increased from 24°C to 28–34°C. In our study, heat-stressed pigs reduced their feed intake, and most strongly during the first HS cycle (662 g/d, approximately 25% between TN1 and HS2).

A reduction in feed intake reduces metabolic heat production in two ways. First, regardless of meal type and size, the postprandial response in mammals is characterized by a 25–50% increase in metabolic rate that usually returns to normal values approximately 6–10 h after eating (Secor, 2009). As proposed by Rauw et al. (1999) and further developed by Speakman and Król (2010), the significant impact of the heat increment of feeding on internal heat production may set an upper central limit to the assimilation of feed resources during periods of increased energy demand, such as lactation. Similarly, when ambient temperature rises, internal heat production can be significantly reduced by reducing feed intake. The broken-line pattern that described increased intensity of behavioral and physiological coping behavior when environmental temperatures increased in the study of Aarnink et al. (2006), also described a decrease in total heat production and voluntary feed intake (Collin et al., 2001; Huynh et al., 2005). Thus, at a certain threshold, pigs linearly reduce internal heat production and feed intake when temperature increases; the threshold for reducing heat production is about one °C lower than the threshold at which voluntary feed intake is reduced, indicating that other physiological processes also play a role (Banhazi et al., 2008).

Second, in response to a decrease in feed intake and as a mechanism to further reduce heat production, key metabolic functions, including tissue growth, decline in heat stressed animals (Baumgard and Rhoads, 2013). For example, growth rate reduced by 11.9% when ambient temperature increased from 24°C to 28–34°C in the study of Hyun et al. (1998), by 16.3% for pigs kept at a hot, diurnal temperature between 22.5 and 35°C compared to pigs kept at a constant, thermoneutral temperature of 20°C in the study of Lopez et al. (1991), by 46% for pigs of 70 kg between 21 and 32°C in the study of Serres (1992), which was consistent with results of Huynh et al. (2005), and by 47% for pigs of 35 kg at 35°C in the study of Pearce et al. (2013). In our study, growth dropped during HS periods, and most strongly during the first HS cycle (529 g/d, nearly 60% between TN1 and HS2).

**Figure 8:**
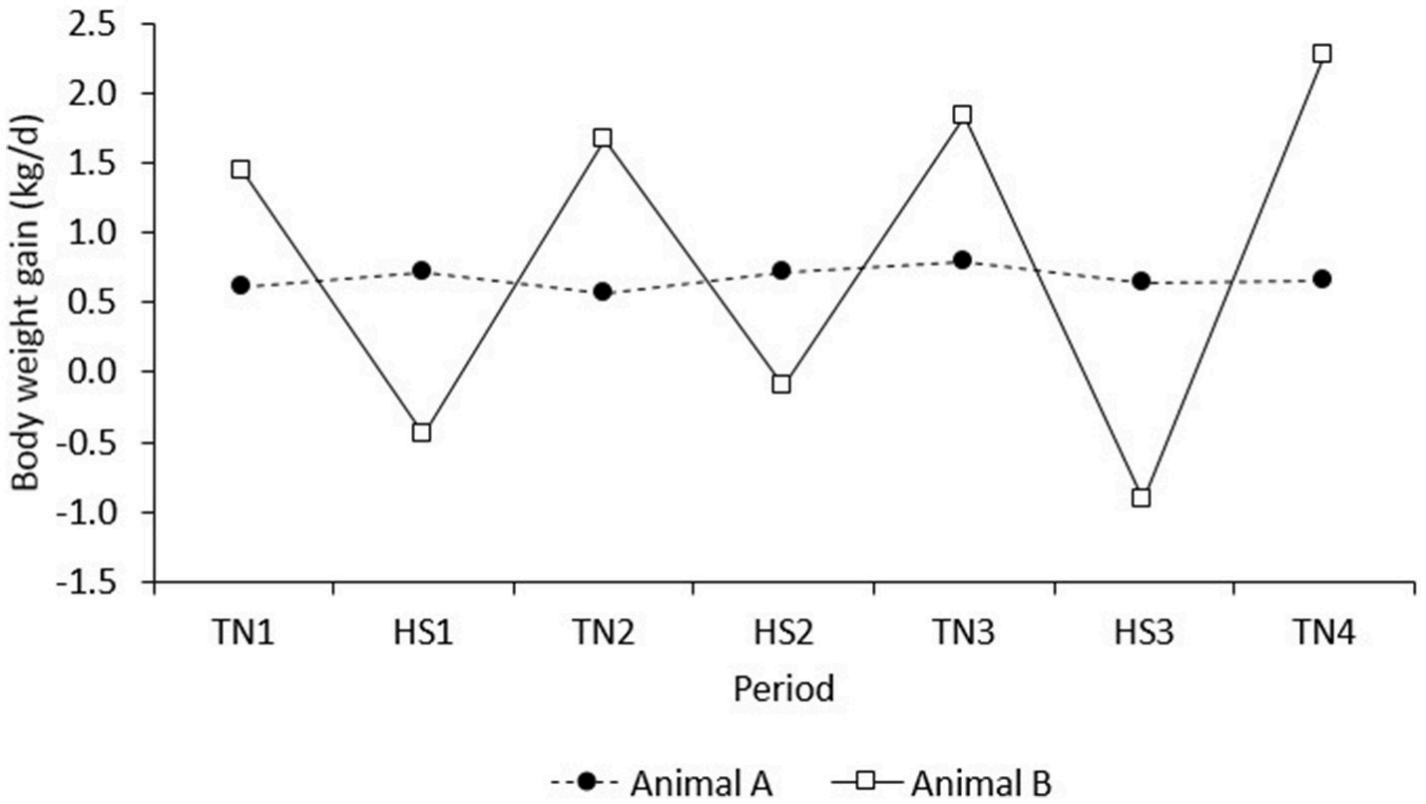
BWG of two extreme examples of individual observations on pigs A and B that depict the negative correlation between BWG in period n with that in period n + 1.

The results of the present study demonstrate that, after correction for line, diet, and room, pigs with higher feed intake in TN conditions maintained a higher FI in HS. However, the results also indicate that pigs with higher growth rates in TN had *lower* growth rates in a subsequent HS challenge. In contrast, the correlation of BWG in the adaptation period (d 1–8) with BWG in period TN1 was positive and not significant (*r* = 0.08, *P* = 0.46; results not presented). This suggests that high producing animals in TN conditions were less robust to HS, whereas those robust to HS showed a trade-off with production under TN conditions. An example of two extreme animals with high vs. low production in TN that depicts this relationship is given in Figure 8. Animal A has low growth in TN, but was clearly more robust to HS, whereas animal B has high growth in TN, but was less robust to HS. This observation supports literature that suggests that environmental sensitivity increases with selection for high production (e.g., Kolmodin et al., 2003; Knap and Su, 2008).

Because of space limitations, our study did not include control line animals of all line and diet combinations that were not subjected to HS at any time. Therefore, our study specifically investigated the response to exposure to heat stress compared with periods of neutral temperatures and the repeatability of the response. Our design did not allow for the evaluation of the deviation in the trend of growth and (cumulative) feed intake over time compared to animals housed in a thermoneutral environment. Therefore, in the present experiment it was not possible to evaluate the full extent of, for example, compensatory growth and consequently the consequences of repeated exposure to heat stress for the time to reach slaughter weight. Given that BWG was similar in all TN periods and the adaptation period (with the exception of commercial pigs, which showed a higher growth rate in TN7), it can be expected that animals were not able to fully recover from lost growth in the three HS periods and therefore that final body weight must have been depressed compared to a control environment.

Cruzen et al. (2015) indicate that HS (32°C) during the finishing period resulted in reduced BWG, BW, and HCW, but did not affect LEA compared with barrows housed in TN conditions (21°C). In addition, although pigs and other species gain more adipose tissue than energetically predicted for their reduced level of feed intake during HS, their carcasses were leaner than those of pigs in TN (Pearce et al., 2013; Cruzen et al., 2015). In the study by Campbell and Taverner (1988), *ad libitum* fed pigs between 9 and 20 kg kept at 32°C had lower growth rates and had a slightly lower proportion of body-fat than those at 14°C. Nienaber and Brown-Brandl (2009) indicated that protein content was maximal and fat content minimal at 30°C while the reverse was true at 15°C. This is supported by our results. Because our experiment did not include a control line that was not subjected to HS, it is not possible to compare the direct effect of HS on slaughter traits. However, our results indicate that animals that were more affected by HS had smaller carcasses and were leaner, without a significant effect on loin eye area. The results indicate that this is mediated through a drop in FI during HS.

### Effect of heat stress on feed efficiency

Feed is the major input to pork production and accounts for more than 65% of all production expenses. Thus, the influence of HS on feed efficiency is of major importance. Feed efficiency is determined by the balance between feed intake and product output. Its most common measure is feed conversion ratio (FCR), which is estimated as FI/BWG, or feed conversion efficiency (FCE), which is the inverse of FCR, estimated as BWG/FI. Higher values of FCE indicate that less feed is needed per unit of growth. Thus, animals with high FCE are more feed efficient. Animals that grow faster also eat more feed, however, BWG accounts for only a portion of the total feed intake. Because it is generally observed that, with faster growth, feed requirements for functions other than growth do not increase proportionally, a positive correlation is generally observed between genetic potential for growth performance and FCE (i.e., a dilution of maintenance requirements). As a consequence, selection for faster growth results in more feed efficient animals when evaluated based on FCE, however, it also results in animals with larger mature size and higher mature maintenance requirements, and therefore greater feed requirements of the breeding herd, which is generally considered undesirable (Crews, 2005). In addition, FCE is defined as a ratio. With direct selection for FCE, the relative selection weights placed on BWG vs. FI depend on selection intensity, along with genetic parameters, and since selection intensity may not be equal for males and females, response to selection in future generations is unpredictable and less than optimal (Gunsett, 1984). These shortcomings of FCR or FCE are solved using an alternative measure of feed efficiency, RFI, first proposed by Koch et al. in 1963. In contrast to FCE, RFI is calculated from a model that allocates total FI to not only BWG, but also to maintenance requirements and, when available, fatness. It is not defined as a ratio and is phenotypically (but, as described by Kennedy et al., 1993, not necessarily genetically) independent of growth and body size (metabolic body weight). Because RFI derived by phenotypic regression depends strongly on the environmental correlation between FI and the component traits, in the present study, the influence of HS on RFI was calculated as a deviation from that expected under normal TN conditions.

In most cases, FCE and FCR can be used interchangeably since they are each other's exact inverse, but this is only true if individuals have positive growth. When growth is zero, FCR is undefined. In addition, when growth is *negative*, which is economically undesirable, both FCR and FCE become negative (note that FI cannot be negative and is positive unless an animal is moribund). Averaging these negative observations in a population in which some animals gain weight and others lose weight, lowers not only the average FCE (a lower efficiency), becoming less desirable, but also the average FCR (reflecting a higher efficiency), becoming *more* desirable. This demonstrates that FCR is not a valid measure of feed efficiency in a population that includes animals that lose weight. Therefore, although a large amount of studies report on FCR in conditions where animals lose weight, in the present study, feed efficiency was represented by FCE.

The results of the present study indicate an apparent discrepancy between the two efficiency measures FCE and RFI: Figure 6 shows that the efficiency measures do not show the same trend. Based on RFI (Figure 6A), pigs are more feed efficient during HS than during TN. However, whereas FCE in low and high RFI pigs indeed appeared to be higher in HS than in TN (this was significant only compared with TN3), in the commercial line, HS resulted in a *decrease* in FCE (*higher* feed efficiency) but also in a decrease in FCE (*lower* feed efficiency). This is not as expected since measures of RFI and FCR are generally reported to be strongly positively correlated (and thus strongly negatively correlated with FCE; Crews, 2005). The discrepancy observed in the present study can be explained when comparing Figures 6A,B. As aforementioned, Figure 6A depicts only part of the relationship of FI with energy sinks by presenting the relationship between FI and BWG, but not that with metabolic body weight and fatness. The regression line depicted in Figure 6A was calculated from model (1) in a TN environment and differs from a regression calculated by regressing FI on BWG only. Figure 6A shows that during HS, a larger number of observations (open dots) fall below the regression line, indicating that, overall, animals during HS consumed less feed than expected based on their levels of (now reduced) growth and were consequently more feed efficient. Put in another way, the higher efficiency based on RFI during HS was caused by animals maintaining higher levels of growth than expected based on their (now reduced) feed intake. Indeed, Table 3 indicates that the overall feed requirements (the intercept) were higher during TN than during HS. Given that basal metabolic rate is hypothesized to increase during HS in response to deployment of thermoregulatory mechanisms, this may not be immediately expected. However, HS has also been shown to result in important shifts in post absorptive metabolism (Baumgard and Rhoads, 2013). Pearce et al. (2013) indicated that HS markedly reduced growth in pigs, but that HS pigs gained more weight than pair-fed controls in TN, which may explain why they were found to be more feed efficient based on RFI in the present study. Figure 6A furthermore shows that RFI is not affected by whether gains are positive or negative, such that observations on animals with negative gains (which is clearly undesirable) that fall below the regression line are considered equally feed efficient (i.e., desirable) as observations with similar regression errors on animals that have positive gains. Also, Figure 6B shows that, during HS (open dots), animals with positive gains tend to have a higher FCE than during TN. Indeed, the correlation between RFI and FCE, adjusted for the effects of period, line, diet, and room, was negative and highly significant (*r* = −0.55, *P* < 0.0001; animals with lower RFI and higher FCE are more feed efficient). However, in contrast to RFI, FCE *does* penalize for negative gains because individual negative FCE observations reduce the average group FCE, and these negative observations only appear in animals that lose weight, during HS.

Whereas animals that eat less than expected, even while losing body weight, may be of interest from a biological perspective, negative gains always seriously affect farm profits since they result in increased time to slaughter and increased fixed costs related to time on farm. Therefore, the negative impact of HS on feed efficiency is more correctly represented by FCE than RFI. It can then be concluded that the results of the present study indicate that 4-d cycles of HS in commercial fast growing lean pigs resulted in increased feed efficiency during these cycles from a biological perspective, however, economic production efficiency was greatly reduced.

### Effect of fiber content on response to heat stress

Since pigs are omnivores, they can consume and utilize a small amount of fibrous feedstuffs to convert into high quality animal protein. High inclusion of co-product feedstuffs from human food production or the biofuel industries is financially attractive. Inclusion of fibrous co-products may possibly affect gut health and pig behavior in a positive way, increase satiety, and overall improve animal well-being (Lindberg, 2014). However, in addition to concerns about variability in nutritional composition, nutrient quality and food safety, energy requirements for and therefore the heat liberated from digestion, absorption, and assimilation of diets that contain a higher relative content of fiber is much greater than with traditional low-fiber diets. For example, Jørgensen et al. (1996) estimated that heat production as a proportion of ME increased from 0.57 to 0.63 when increasing the dietary fiber (DF) content from 59 to 268 DF/kg diet DM. As a consequence, pigs fed a high fiber diet may be more susceptible to environmental HS.

The results of our study indicate that commercial pigs fed a high fiber diet had a lower feed intake than pigs fed a regular low-fiber diet, while no differences were found for BWG, RFI, or FCE. As reviewed by Noblet and Le Goff (2001), pigs can digest DF to a reasonable extent, but, depending on the botanical origin, its digestibility is much more variable and significantly lower than that of other nutrients such as starch, sugars, fat, and protein. In addition, since digestibility increases with age and live weight, DF does not have uniform nutritional effects. More specifically, co-products from ethanol plants or “distillers dried grains with solubles” (DDGS), which was the origin of DF in the diets of the present study, also reduces digestibility of dry matter and the digestibility of energy in DDGS. Stein and Shurson (2009), reviewing studies on the inclusion of corn DDGS in grower-finisher diets, reported that average daily gain was improved in one study, reduced in six studies, and not affected in 18 studies, FI was increased in two studies, reduced in six studies, and not affected in 15 studies, and FCE was improved in four studies, reduced in five studies, and not affected in 16 studies. According to the authors, a reduction in FI may have resulted from reduced palatability of diets containing DDGS (Stein and Shurson, 2009), however, Weber et al. (2015) fed up to 60% DDGS with minimal impact on performance. Alternatively, a reduction in FI may have resulted from incorrect formulation of the diets or from a limited gut capacity to assimilate a higher bulk mass (Beaulieu et al., 2009). Because pigs eat to satisfy their energy requirements, it is generally observed that lower digestible energy content with inclusion of additional fiber is compensated by an increase in voluntary feed intake (Low, 1985). However, in our study the diets had very similar net energy content. Our results did not detect a diet by climate interaction and therefore no indication that pigs fed a high fiber diet are more susceptible to HS. The 20% inclusion of DDGS in our study may not have been large enough to elicit the anticipated diet by climate interaction.

### Effect of selection for high lean tissue growth rate and feed efficiency on response to heat stress

Since metabolic functions are directly related to metabolic heat production, the animal's genetic potential for production traits can be expected to influence its susceptibility to HS. For example, due to the large amount of water contained in lean tissue, the energy cost for lean deposition is much lower than that of fat deposition, however, lean tissue is associated with increased maintenance requirements due to high protein turnover rates. As a result, fat pigs produce less heat per unit metabolic size than lean pigs (Sundstøl et al., 1979; Tess et al., 1984). Brown-Brandl et al. (2004) concluded that fasting heat production increased by 18.1% between 1984 and 2002 as a result of increased lean tissue accretion rates. This suggests that pigs with high potential of lean accretion may be more susceptible to HS. Indeed, a meta-analysis by Renaudeau et al. (2011) indicated that the effect of increased ambient temperature on growth and feed intake was greater in more contemporary literature, suggesting that modern genotypes may be more sensitive to HS than older genotypes with lesser growth potential. Nienaber et al. (1997) reported a reduction of 4°C of the upper critical temperature for pigs of newer genetics. In our study, commercial pigs, that were considerably leaner and grew considerably faster in TN than pigs from the low and high RFI lines, had a considerably larger drop in BWG from TN to HS climates than pigs from the low and high RFI lines. Residual growth rate in TN vs. HS, estimated by the residual e_i_ by switching FI and BWG in model (1), indicated that commercial pigs grew about 0.127 kg/d more than expected in TN but 0.382 kg/d less than expected in HS, while the low and high RFI lines grew 0.011 and 0.130 kg/d less than expected in TN but 0.062 and 0.130 kg/d more than expected in HS, respectively (results not presented). The drop in BWG in the commercial line was so large that their superiority in growth rate over the RFI lines was no longer maintained in HS conditions. However, in other words, growth performance of commercial pigs during HS was still as good as that of both RFI lines. During TN, superior growth rate of the commercial pigs was responsible for their superior FCE (i.e., economic efficiency), however, because the reduction in FI during HS was not significantly different between the lines, commercial pigs also lost their superiority in FCE and became less efficient than the low and high RFI lines under HS. These results indicate that high lean tissue growth rate in commercial pigs negatively influences their robustness to HS. Table 3 indicates that commercial pigs required more feed per unit of BWG (the intercept) compared to pigs of the low and high RFI lines, which explains their higher RFI estimates (i.e., biological *in*efficiency compared to those lines). During HS, feed requirements for BWG were also higher in commercial pigs than in pigs from the low and high RFI lines.

Decreased resilience to HS as a result of selection for high lean tissue growth rate may be to some extent ameliorated by genetic selection for increased feed efficiency, since pigs with a greater feed efficiency have lower basal metabolic rates. For example, Barea et al. (2010) and Renaudeau et al. (2013) showed that pigs selected for low RFI exhibited lower heat production, resulting from a lower basal metabolic rate, than pigs selected for high RFI. This suggests that pigs from the low RFI line, i.e., efficient pigs, may be less susceptible to HS than pigs from the line selected for high RFI. However, results of the presents study show that low RFI pigs actually had a somewhat *larger* (albeit non-significant) drop in BWG in HS1 and HS2. Although they did not report changes in BWG or in RFI, Renaudeau et al. (2013) observed similar changes in energy metabolism (heat production, maintenance requirements, fasting heat production, thermic effect of feeding, and activity heat production) for pigs selected for low and high RFI during a thermal acclimation period to 32°C, suggesting that HS impacted energy metabolism for pigs from these two lines to a similar extent. They also showed that sensible heat loss and water consumption was greater in pigs from the high RFI line compared to pigs from the low RFI line. It is possible that such differences were responsible for the larger (but non-significant) drop in BWG in the low RFI line compared with the high RFI line in the present study. This, indeed, was observed in the study of Bordas and Minvielle (1997), in which broiler chickens from a high RFI line had a lower reduction in egg number during HS; they suggested that their better adaptation to HS may be due partly to a higher capacity for heat dissipation.

## Synthesis and conclusion

The farm animal of the future is described as robust, adapted, and healthy (Mormède et al., 2011), i.e., having “the ability to combine a high production potential (growing or reproductive) with resilience to stressors, allowing for unproblematic expression of a high production potential in a wide variety of environmental conditions” (Knap, 2005). Globally, more than 50% of total meat and 60% of milk produced originates from tropical and subtropical areas, where resilience to the hot climate is one of the main limiting factors of production efficiency (Renaudeau et al., 2012). In addition, higher temperatures and severe and widespread droughts resulting from global warming are expected in the next 30–90 years (Dai, 2013). Several studies predict severe production losses if current operations are not modified to reflect the predicted shift in climate (Gaughan et al., 2009). For example, St-Pierre et al. (2003) estimated that without heat abatement, total losses across livestock animal classes by US industries would average $2.4 billion annually. Therefore, adaptation of livestock to hot climates is of increasing importance to livestock production.

Our results support earlier observations that HS in pigs results in reduced FI and BWG. The drop in FI results in leaner pigs that generate smaller carcasses at slaughter. Our results furthermore indicate that animals with a larger drop in BWG between TN1 and HS1 tended to have a larger drop between TN2 and HS2, and those with a larger drop between TN2 and HS2 also had a larger drop between TN3 and HS3. This suggests that there is some repeatability in robustness to HS. Although feed efficiency in response to HS improved biologically (efficiency measured as RFI), economically it was deteriorated (efficiency measured as FCE), in particularly in the commercial line.

Management strategies to alleviate HS in farm animals were reviewed by St-Pierre et al. (2003) and Renaudeau et al. (2012), and include improvement of the design of facilities, reducing stocking density, reducing manipulation of animals and other additional stressors, and improving feeding strategies and composition. Because including dietary fiber results in a concomitant increase in heat liberated from digestion, absorption, and assimilation, pigs fed a high fiber diet are expected to be more susceptible to environmental HS. Our results using diets that included fibrous corn DDGS did, however, not support this; the impact of the high fiber diet may have been reduced by the similar net energy content of the diets, and the observation that FI in commercial pigs eating high fiber diets was actually lower than pigs eating regular diets.

Our results support the observation that genetic make-up directly influences robustness to HS through differences in metabolic rate resulting from the level of lean tissue growth rate. Pigs from the commercial line, which had considerably faster lean tissue growth rates than pigs from the low and high RFI lines in TN, had the largest drop in BWG between TN and HS. This drop was to such a degree that commercial pigs, during HS, lost their superiority in BWG over the low and high RFI pigs. Nonetheless, when evaluated over the entire growth period, pigs from the commercial line clearly had a more desirable production performance. In addition, independent of line, pigs with very high production potential in TN were less robust to HS. This observation supports literature that suggests that environmental sensitivity increases with selection for high production levels. Long-term selection for feed efficiency under TN conditions does not appear to have improved heat tolerance.

Misztal (2017) indicates that to date there is not a high level of interest by commercial breeding companies for selection for HS in dairy cattle. However, selection for robustness to HS may be more advantageous for pig breeding companies because of a shorter generation interval. Our results support the necessity to review breed choice and genetic selection objectives for improved robustness to climates with recurrent periods of HS. As reviewed by Renaudeau et al. (2012), this may involve selecting for heat tolerance in commercial pig lines or by introgression of heat adaptation genes from local breeds into a commercial line. Because differences between breeds in response to HS appeared more variable for BWG than for FI, based on the results of the present study, pigs of interest as selection candidates are those that are able to maintain high growth rates under HS. Our results also showed that response in growth to HS was repeatable over subsequent 4-d cycles of HS, which suggests the potential for inclusion of the response in BWG to a 4-d HS cycle in the breeding index. The best performing animals are likely those that are not highly superior for growth in TN.

## Author contributions

Funding was obtained by LB, JD, JP, NG, and SMLo. LB conducted the live-phase of the experiment. SMLo performed the carcass evaluations after slaughter. Data were analyzed by EM and WR. WR wrote the manuscript. All authors discussed and corrected the drafts and accepted the final version of the manuscript.

### Conflict of interest statement

The authors declare that the research was conducted in the absence of any commercial or financial relationships that could be construed as a potential conflict of interest.

## References

[B1] Aarnink A. J. A. Schrama J. W. Heetkamp M. J. W. Stefanowska J. Huynh T. T. T. (2006). Temperature and body weight affect fouling of pig pens. J. Anim. Sci. 84, 2224–2231. 10.2527/jas.2005-521 16864884

[B2] Banhazi T. Aarnink A. Thuy H. Pedersen S. Hartung J. Payne H. (2008). Review of Issues Related to Heat Stress in Intensively Housed Pigs. Livestock Environment VIII. ASABE Publication Number 701P0408 Available online at: http://elibrary.asabe.org/azdez.asp?AID=25578&t=2

[B3] Barea R. Dubois S. Gilbert H. Sellier P. Van Milgen J. Noblet J. (2010). Energy utilization in pigs selected for high and low residual feed intake. J. Anim. Sci. 88, 2062–2072. 10.2527/jas.2009-2395 20154162

[B4] Baumgard L. H. Rhoads R. P. (2013). Effects of heat stress on postabsorptive metabolism and energetics. Annu. Rev. Anim. Biosci. 1, 311–337. 10.1146/annurev-animal-031412-103644 25387022

[B5] Beaulieu A. D. Williams N. H. Patience J. F. (2009). Response to dietary digestible energy concentration in growing pigs fed cereal grain-based diets. J. Anim. Sci. 87, 965–976. 10.2527/jas.2007-0834 19098234

[B6] Berman A. Meltzer A. (1973). Critical temperatures in lactating dairy cattle: a new approach to an old problem. Int. J. Biometeorol. 17, 167–176.

[B7] Black J. L. Mullan B. P. Lorschy M. L. Giles L. R. (1993). Lactation in the sow during heat stress. Livest. Prod. Sci. 35, 153–170. 10.1016/0301-6226(93)90188-N

[B8] Bordas A. Minvielle F. (1997). Réponse à la chaleur de poules pondeuses issues de lignées sélectionnées pour une faible (R-) ou forte (R+) consommation alimentaire résiduelle. Genet. Sel. Evol. 29, 279–290. 10.1186/1297-9686-29-2-279

[B9] Brown-Brandl T. M. Nienaber J. A. Turner L. W. Yen J. T. (2000). Manual and thermal induced feed intake restriction on finishing barrows: I: effects on growth, carcass composition, and feeding behavior. Transac. ASAE 43, 987–992. 10.13031/2013.2996

[B10] Brown-Brandl T. M. Nienaber J. A. Xin H. Gates R. S. (2004). A literature review of swine heat production. Trans. ASAE 47, 259–270. Available online at: https://experts.illinois.edu/en/publications/a-literature-review-of-swine-heat-production

[B11] Cai W. Casey D. S. Dekkers J. C. M. (2008). Selection response and genetic parameters for residual feed intake in Yorkshire swine. J. Anim. Sci. 86, 287–298. 10.2527/jas.2007-0396 17998435

[B12] Campbell R. G. Taverner M. R. (1988). Relationships between energy intake and protein and energy metabolism, growth and body composition of pigs kept at 14 or 32°C from 9 to 20 kg. Livest. Prod. Sci. 18, 289–303. 10.1016/0301-6226(88)90037-1

[B13] Collin A. Van Milgen J. Le Dividich J. (2001). Modelling the effect of high, constant temperature on food intake in young growing pigs. Anim. Sci. 72, 519–527. 10.1017/S1357729800052048

[B14] Crews D. H. Jr. (2005). Genetics of efficient feed utilization and national cattle evaluation: a review. Genet. Mol. Res. 4, 152–165. Available online at: http://www.funpecrp.com.br/gmr/year2005/vol2-4/pdf/gmr0124.pdf 16110437

[B15] Cruzen S. M. Boddicker R. L. Graves K. L. Johnson T. P. Arkfeld E. K. Baumgard L. H.. (2015). Carcass composition of market weight pigs subjected to heat stress in utero and during finishing. J. Anim. Sci. 93, 2587–2596. 10.2527/jas.2014-8347 26020353

[B16] Curtis S. (1983). Perception of thermal comfort by farm animals, in Farm Animal Housing and Welfare, eds Baxter S. H. Baxter M. R. MacCormack J. A. C. (Dordrecht: Martinus Nijhoff Publishers), 59–66.

[B17] Dai A. (2013). Increasing drought under global warming in observations and models. Nat. Clim. Change 3, 52–58. 10.1038/nclimate1633

[B18] Eissen J. J. Kanis E. Kemp B. (2000). Sow factors affecting voluntary feed intake during lactation. Livest. Prod. Sci. 64, 147–165. 10.1016/S0301-6226(99)00153-0

[B19] Gaughan J. Lacetera N. Valtorta S. E. Khalifa H. H. Hahn L. Mader T. (2009). Response of domestic animals to climate challenges, in Biometerology for Adaptation to Climate Variability and Change, eds Ebi K. L. Burton I. McGregor G. (Dordrecht: Springer), 131–170.

[B20] Gunsett F. C. (1984). Linear index selection to improve traits defined as ratios. J. Anim. Sci. 59, 1185–1193.

[B21] Hyun Y. Ellis M. Riskowski G. Johnson R. W. (1998). Growth performance of pigs subjected to multiple concurrent environmental stressors. J. Anim. Sci. 76, 721–727. 10.2527/1998.763721x 9535330

[B22] Huynh T. T. T. Aarnink A. J. A. Verstegen M. W. A. Gerrits W. J. J. Heetkamp M. J. W. Kemp B.. (2005). Effects of increasing temperatures on physiological changes in pigs at different relative humidities. J. Anim. Sci. 83 1385–1396. 10.2527/2005.8361385x 15890816

[B23] Jørgensen H. Zhaoy X. Q. Eggum B. O. (1996). The influence of dietary fibre and environmental temperature on the development of the gastrointestinal tract, digestibility, degree of fermentation in the hind-gut and energy metabolism in pigs. Br. J. Nutr. 75, 365–378. 8785211 10.1079/bjn19960140

[B24] Kadzere C. T. Murphy M. R. Silanikove N. Maltz E. (2002). Heat stress in lactating dairy cows: a review. Livestock Prod. Sci. 77, 59–91. 10.1016/S0301-6226(01)00330-X

[B25] Kennedy B. W. Van der Werf J. H. J. Meuwissen T. H. E. (1993). Genetic and statistical properties of residual feed intake. J. Anim. Sci. 71, 3239–3250. 8294275 10.2527/1993.71123239x

[B26] Knap P. W. (2005). Breeding robust pigs. Aust. J. Exp. Agr. 45, 763–773. 10.1071/EA05041

[B27] Knap P. W. Su G. (2008). Genotype by environment interaction for litter size in pigs as quantified by reaction norms analysis. Animal 2, 1742–1747. 10.1017/S1751731108003145 22444079

[B28] Koch R. M. Swiger L. A. Chambers D. Gregory K. E. (1963). Efficiency of feed use in beef cattle. J. Anim. Sci. 22, 486–494. 10.2527/jas1963.222486x

[B29] Kolmodin R. Strandberg E. Jorjani H. Danell B. (2003). Selection in the presence of a genotype by environment interaction: response in environmental sensitivity. Anim. Sci. 76, 375–385. 10.1017/S1357729800058604

[B30] Lara L. J. Rostagno M. H. (2013). Impact of heat stress on poultry production. Animals 3, 356–369. 10.3390/ani3020356 26487407 PMC4494392

[B31] Le Dividich J. Noblet J. Herpin P. Van Milgen J. Quiniou N. (1998). Thermoregulation, in Progress in Pig Science, eds Wiseman J. Varley M. A. Chadwick J. P. (Nottingham: Nottingham University Press), 229–263.

[B32] Lindberg J. E. (2014). Fiber effects in nutrition and gut health in pigs. J. Anim. Sci. Biotechn. 5:15. 10.1186/2049-1891-5-15 24580966 PMC3975931

[B33] Lopez J. Jesse G. W. Becker B. A. Ellersieck M. R. (1991). Effects of temperature on the performance of finishing swine: 1. Effects of a hot, diurnal temperature on average daily gain, feed intake, and feed efficiency. J. Ani. Sci. 69, 1843–1849. 10.2527/1991.6951843x 2066294

[B34] Low A. G. (1985). Role of dietary fiber in pig diets, in Recent Advances in Animal Nutrition, eds Haresign W. Cole D. J. A. (London: Butterworths), 87–112.

[B35] Millward D. J. Garlick P. J. (1976). The energy cost of growth. P. Nutr. Soc. 35, 339–349. 800657 10.1079/pns19760054

[B36] Misztal I. (2017). Resilience and lessons from studies in genetics of heat stress. J. Anim. Sci. 2017:95 10.2527/jas.2016.0953 28464095

[B37] Mormède P. Foury A. Terenina E. Knap P. W. (2011). Breeding for robustness: the role of cortisol. Animal 5, 651–657. 10.1017/S1751731110002168 22439987

[B38] National Research Council (1998). Nutrient Requirements of Swine. 10th Revised Edn. Subcommittee on Swine Nutrition, Committee on Animal Nutrition, National Research Council. Washington, DC: National Academy Press.

[B39] Naya D. E. Bacigalupe L. D. (2009). Metabolic constraints to resource allocation, in Resource Allocation Theory Applied to Farm Animal Production, ed Rauw W. M. (Wallingford, CT: CAB International), 61–71.

[B40] Nienaber J. A. Brown-Brandl T. M. (2009). Heat stress effects on growing-finishing swine, in 25th Annual Caroline Swine Nutrition Conference, 25–35. Available online at: https://naldc.nal.usda.gov/download/46453/PDF

[B41] Nienaber J. A. Hahn G. L. Eigenberg R. A. Korthals R. L. Yen J. T. Harris D. L. (1997). Genetic and heat stress interaction effects on finishing swine, in Proceedings of the International Livestock Environment Symposium IV (Coventry), 1017–1023.

[B42] Noblet J. Le Goff G. (2001). Effect of dietary fibre on the energy value of feeds for pigs. Anim. Feed Sci. Tech. 90, 35–52. 10.1016/S0377-8401(01)00195-X

[B43] Pearce S. C. Gabler N. K. Ross J. W. Escobar J. Patience J. F. Rhoads R. P.. (2013). The effects of heat stress and plane of nutrition on metabolism in growing pigs. J. Anim. Sci. 91, 2108–2118. 10.2527/jas.2012-5738 23463563

[B44] Pollmann D. S. (2010). Seasonal Effects on Sow Herds: Industry Experience and Management Strategies. Des Moines, IA: Midwest American Society of Animal Science Available at: https://vimeo.com/46531288

[B45] Quiniou N. Dubois S. Noblet J. (2000). Voluntary feed intake and feeding behaviour of group-housed growing pigs are affected by ambient temperature and body weight. Livest. Prod. Sci. 63, 245–253. 10.1016/S0301-6226(99)00135-9

[B46] Rauw W. M. Gomez-Raya L. (2015). Genotype by environment interaction and breeding for robustness in livestock. Front. Genet. 6:310. 10.3389/fgene.2015.00310 26539207 PMC4612141

[B47] Rauw W. M. Knap P. W. Verstegen M. W. A. Luiting P. (2002). Food resource allocation patterns in lactating females in a long-term selection experiment for litter size in mice. Genet. Sel. Evol. 34:83. 10.1186/1297-9686-34-1-83 11929626 PMC2705424

[B48] Rauw W. M. Luiting P. Beilharz R. G. Verstegen M. W. A. Vangen O. (1999). Selection for litter size and its consequences for the allocation of feed resources: a concept and its implications illustrated by mice selection experiments. Livest. Prod. Sci. 60, 329–342. 10.1016/S0301-6226(99)00104-9

[B49] Renaudeau D. Collin A. Yahav S. De Basilio V. Gourdine J. L. Collier R. J. (2012). Adaptation to hot climate and strategies to alleviate heat stress in livestock production. Animal 6, 707–728. 10.1017/S1751731111002448 22558920

[B50] Renaudeau D. Frances G. Dubois S. Gilbert H. Noblet J. (2013). Effect of thermal heat stress on energy utilization in two lines of pigs divergently selected for residual feed intake. J. Anim. Sci. 91, 1162–1175. 10.2527/jas.2012-5689 23296816

[B51] Renaudeau D. Gourdine J. L. St-Pierre N. R. (2011). A meta-analysis of the effects of high ambient temperature on growth performance of growing-finishing pigs. J. Anim. Sci. 89, 2220–2230. 10.2527/jas.2010-3329 21297065

[B52] Saxton C. (1981). Effects of severe heat stress on respiration and metabolic rate in resting man. Aviat. Space Envir. Md. 52, 281–286. 7247898

[B53] Schrama J. W. Van der Her W. Gorssen J. Henken A. M. Verstegen M. W. A. Noordhuizen J. P. T. M. (1996). Required thermal thresholds during transport of animals. Vet. Quart. 18, 90–95. 10.1080/01652176.1996.9694624 8903140

[B54] Secor M. (2009). Specific dynamic action: a review of the postprandial metabolic response. J. Comp. Physiol. B 179, 1–56. 10.1007/s00360-008-0283-7 18597096

[B55] Serres H. (1992). Manual of Pig Production in the Tropics. Wallingford, CT: CAB International.

[B56] Smith R. M. Gabler N. K. Young J. M. Cai W. Boddicker N. J. Anderson M. J.. (2011). Effects of selection for decreased residual feed intake on composition and quality of fresh pork. J. Anim. Sci. 89, 192–200. 10.2527/jas.2010-2861 20817860

[B57] Speakman J. R. Król E. (2010). The heat dissipation limit theory and evolution of life histories in endotherms—time to dispose of the disposable soma theory? Integr. Comp. Biol. 50, 793–807. 10.1093/icb/icq049 21558242

[B58] Stein H. H. Shurson G. C. (2009). Board-invited review: the use and application of distillers grains with solubles in swine diets. J. Anim. Sci. 87, 1292–1303. 10.2527/jas.2008-1290 19028847

[B59] St-Pierre N. R. Cobanov B. Schnitkey G. (2003). Economic losses from heat stress by US livestock industries. J. Dairy Sci. 86(E. Suppl.), E52–E77. 10.3168/jds.S0022-0302(03)74040-5

[B60] Sundstøl F. Standal N. Vangen O. (1979). Energy metabolism in lines of pigs selected for thickness of backfat and rate of gain. Acta Agric. Scand. 29, 337–345. 10.1080/00015127909435246

[B61] Tess M. W. Dickerson G. E. Nienaber J. A. Ferrell C. L. (1984). The effects of body composition on fasting heat production in pigs. J. Anim. Sci. 58, 99–110. 10.2527/jas1984.58199x 6698908

[B62] USDA (2015). Climate change, global food security, and the U.S. food system, in U.S. Global Change Research Program. U.S. Department of Agriculture Available online at: https://www.usda.gov/oce/climate_change/FoodSecurity2015Assessment/FullAssessment.pdf

[B63] Weber E. K. Stalder K. J. Patience J. F. (2015). Wean-to-finish feeder space availability effects on nursery and finishing pig performance and total tract digestibility in a commercial setting when feeding dried distillers grains with solubles. J. Anim. Sci. 93, 1905–1915. 10.2527/jas2014-8136 26020213

